# Disruption of international trade and its propagation through firm-level domestic supply chains: A case of Japan

**DOI:** 10.1371/journal.pone.0294574

**Published:** 2023-11-27

**Authors:** Hiroyasu Inoue, Yasuyuki Todo

**Affiliations:** 1 Graduate School of Information Science, University of Hyogo, Kobe, Hyogo, Japan; 2 Japan Science and Technology Agency, Kawaguchi, Saitama, Japan; 3 RIKEN Center for Computational Science, Kobe, Hyogo, Japan; 4 Graduate School of Economics, Waseda University, Shinjuku, Tokyo, Japan; 5 Research Institute of Economy, Trade and Industry, Minato, Tokyo, Japan; Bucharest University of Economic Studies: Academia de Studii Economice din Bucuresti, ROMANIA

## Abstract

This study simulates how the disruption of imports from various regions affects the total production of the importer economy. We particularly incorporate the propagation of the economic effect through domestic supply chains using data on more than one million firms and four million supply chain ties in Japan. Our findings are summarized as follows. First, the negative effect of the disruption of intermediate imports grows exponentially as its duration and strength increase due to downstream propagation. Second, the propagation of the economic effect is substantially affected by the network topology of importers, such as the number of importers (affected nodes) and their degree of upstreamness in supply chains, whereas the effect of their degree centrality is heterogeneous depending on their degree of upstreamness. Finally, the negative effect of import disruption can be mitigated by the reorganization of domestic supply chains, even when conducted only among network neighbors. Our findings provide important policy and managerial implications for the achievement of more robust and resilient global supply chains.

## Introduction

The robustness and resilience of global supply chains, i.e., the network of trade in materials and parts for production across countries and industries, are currently threatened [1]. In recent years, global supply chains have frequently been disrupted by coronavirus disease 2019 (COVID-19) [2–5]; natural disasters, such as the Great East Japan earthquake [6–8]; military conflicts, such as the Russo-Ukrainian War [9, 10]; and the trade and investment policies of the United States and its allies and China to “decouple” from one another [11]. The risk of disruption to global supply chains has been rising because pandemics and natural disasters are predicted to occur more frequently in the future due to climate change and seismic cycles [12–16]. In addition, this disruption risk has intensified because of the increasing possibilities of countries “weaponizing” supply chains by restricting trade with particular partners for national security reasons as global geopolitical risks soar [17, 18].

When the imports and exports of a country through global supply chains are disrupted, the resulting economic loss could be quite large because such disruption not only affects importer and exporter firms directly but also affects other firms linked with importers and exporters through domestic supply chains. When the imports of materials and parts are disrupted, importer firms should reduce their level of production due to the shortage of supplies. This production reduction translates into a reduction in the production of importer firms’ domestic suppliers due to the shortage of demand and their domestic customer firms due to the shortage of supply. This upstream and downstream propagation of the effect of supply chain disruptions is theorized in industry- [4, 19, 20] and firm-level models [6, 8].

Therefore, it is vital to understand how substantial the effect of a possible large-scale trade disruption would be, how much this effect would be magnified by propagation through domestic supply chains, and how the robustness and resilience of the domestic economy can be achieved by alleviating propagation. However, several strands of the literature examining the effect of supply chain disruption may not fully answer these questions, as explained in detail in the next section. In short, some studies have focused on interindustry propagation and thus ignored the network complexity of firm-level supply chains, whereas other studies have examined firm-level propagation but not shown the effect on the whole economy.

Given this research gap, this study estimates the effect of the disruption of the imports of inputs from various regions, including China, on the domestic production of Japan while accounting for supply chain propagation. Specifically, we simulate an agent-based model with large-scale data on more than one million firms that contain detailed supply chain information and information on imports and exports at the firm level. Our model and simulations extend those used in the literature focusing on the propagation of domestic economic shocks through domestic supply chains [21–25] by incorporating international trade. To the best of our knowledge, this study is the first to simulate the effect of the disruption of global supply chains on the domestic economy using a model and data at the firm level.

We are particularly interested in the effect of the disruption of imports from China for two reasons. First, China’s share of Japan’s imports of intermediate products in 2021 was 26% [26], indicating the heavy reliance of Japan on China in supply chains. Second, the trade value of products imported between Japan and China has recently shrunk from 28.9 billion dollars in January 2022 to 24.7 billion in January 2023, or by approximately 15% [26], possibly because of the current policies in Japan that restrict the trade of high-tech products with China due to national security concerns [11]. Therefore, once a larger-scale disruption of trade with China occurs, its effect on the Japanese economy can be devastating and thus should be estimated.

To examine how the robustness and resilience of global supply chains can be strengthened, we further analyze how the network topology of affected nodes, i.e., firms importing intermediate goods, influences propagation through the network and the total effect. In addition to measures of centrality and density, which have often been found to be influential in the literature [27, 28], we employ measures of the upstreamness of affected nodes and the loops in which they are involved as possible determinants of propagation. Finally, we investigate the role of the substitutability of suppliers in the resilience of supply chains.

Our findings are summarized as follows. First, the negative effect of the disruption of intermediate imports grows exponentially as its duration and strength increase due to downstream propagation. Second, the propagation of the economic effect is substantially affected by the network topology of importers, such as the number of importers and their degree of upstreamness in supply chains, whereas the effect of their degree centrality is heterogeneous depending on their degree of upstreamness. Finally, the negative effect of import disruption can be mitigated by the reorganization of domestic supply chains, even when conducted only among network neighbors.

## Related literature

Several strands of literature have extensively examined the effect of supply chain disruptions, although there are differences in the methodologies and purposes of these studies compared to the present study.

First, some studies estimate the economic effect of disruptions to global supply chains using theoretical models and industry-level data. For example, [20] develops a multicountry and multiindustry model that combines a dynamic stochastic general equilibrium (DSGE) model and a computable general equilibrium (CGE) model. Then, the above study estimates the effect of reductions in labor and demand due to the COVID-19 pandemic in a particular region on the production of major economies. [4] employs an agent-based model with multiple countries and industries that incorporates the substitution of disrupted supply chains and estimates the economic effect of the lockdown of one region on another. Both studies use international input–output (IO) tables to identify IO linkages across countries and industries and find that the economic effect of lockdown in a region propagates through these IO linkages and thus can be substantial. However, their major shortcoming is that they use IO linkages at the country-industry level and ignore the complexity of supply chains at the firm level, which can aggravate the propagation of economic shocks, as suggested in the network literature [22, 25, 29, 30].

Second, several other studies take an econometric approach to investigate the propagation of foreign shocks due to natural disasters on domestic production through supply chains using firm-level data. This approach is first utilized to examine propagation through domestic supply chains [6, 8] and subsequently extended to international propagation. For example, [7] finds a negative effect of the Great East Japan earthquake due to a shortage of parts and components from Japan. In contrast, [31] uses data covering major firms in the world and their major supply chain partners and find no significant effect of Hurricane Sandy in the US in 2012 on the sales of suppliers or clients outside the US of those firms directly affected by the hurricane. Although this econometric approach can clarify whether and how much a reduction in the supply and demand of foreign firms affects the production of their supply chain partners, it cannot estimate its total effect on the whole economy.

Third, some other studies estimate how imports from China affect the importer economy, particularly its level of employment, using econometric approaches. [32, 33] use industry-level data for the US and find that the level of penetration of imports from China lowers the level of employment in the US manufacturing industry and further find that the negative effect of Chinese imports on US employment propagates through IO linkages and is largely exacerbated. Moreover, [34, 35] apply this framework to Germany and Japan, respectively. Using a similar framework but firm-level data in addition to industry-level data for Japan, [36] analyzes how the effect of imports from China on the sales of Japanese firms propagate upstream (i.e., from importers to their suppliers) and downstream (i.e., from importers to their clients). Although these studies highlight the negative effects of imports from China on domestic manufacturing industries in importer countries, their analytical framework does not reveal how disruptions of imports from China affect domestic production.

Fourth, there is a great deal of social network literature on the relationship between the network topology and diffusion of information and behaviors through the network. The literature often finds the centrality of each node and the density of its ego network as major determinants of diffusion [37–39], as summarized in [40]. We contribute to the literature by incorporating the measures of the degree of upstreamness of each affected node and those loops where it is involved that are calculated from the Helmholtz–Hodge decomposition (HHD) developed by [41–43]. Our finding that the degree of upstreamness of affected nodes in the network plays an important role in diffusion is new in the literature.

Fifth, our analyses are also related to another strand of the network literature that investigates how rewiring or adding new links in response to the removal of nodes or links affects the robustness and resilience of network connectivity [44–46]. We find that connecting firms facing supply disruption with new suppliers among their network neighbors can mitigate the negative effect as much as can connecting with new suppliers chosen from the whole network, thus contributing to the literature.

Finally, the management literature has extensively discussed supply chain resilience. Moreover, the literature often emphasizes the role of adaptation and flexibility within the firm, including the flexible adaptation of products and technologies, and across firms, including interfirm collaboration and flexible substitution of partners [47–50]. Some studies also suggest reconsidering the optimum inventory management mode that may require redundancy to prepare for supply chain disruption [2, 50]. Our findings that suggest the importance of the substitution of partners and inventory management are mostly consistent with these conclusions.

## Materials and method

### Data

This study uses two datasets. The first set comes from the Company Information Database and Company Linkage Database of Tokyo Shoko Research (TSR) for 2020, which contains attributes for most firms, including small and medium-sized enterprises (SMEs) and their major domestic clients and suppliers, in Japan. After removing those firms without sales information, the numbers of firms and supply chain links in the sample are 104,9697 and 4,957,967, respectively. Because the TSR data do not contain the sales of each firm to final consumers and the transaction volume of each supply chain link, we estimate these factors using the IO table of Japan in 2015 [51] so that the aggregate transactions between industries and final consumers according to firm-level estimations match those in the IO table.

The other dataset is the Basic Survey of Japanese Business Structure and Activities (*Kigyo Katsudo Kihon Chosa*, hereafter referred to as the BSJ) collected annually by the Ministry of Economy, Trade and Industry (METI). The BSJ targets firms in Japan with 50 or more employees and initial capital of 30 or more million yen, i.e., relatively large firms. The response rate of the BSJ in 2019 is 78.8%, and the number of respondent firms is 37,162. BSJ data include information on the imports of inputs from and the exports of outputs of firms to broadly classified foreign regions and countries, i.e., Asia, China, Europe, North America, the Middle East, and other regions. Throughout the study, we follow the definition of regions used in the BSJ data and denote East Asia, including China, Southeast Asia, South Asia, and Central Asia as “Asia” and West Asia as “the Middle East.” We combine the TSR data with trade information at the firm level taken from BSJ data, using firm identification numbers for the BSJ that are also included in the TSR data.

### Model

**Fig 1:**
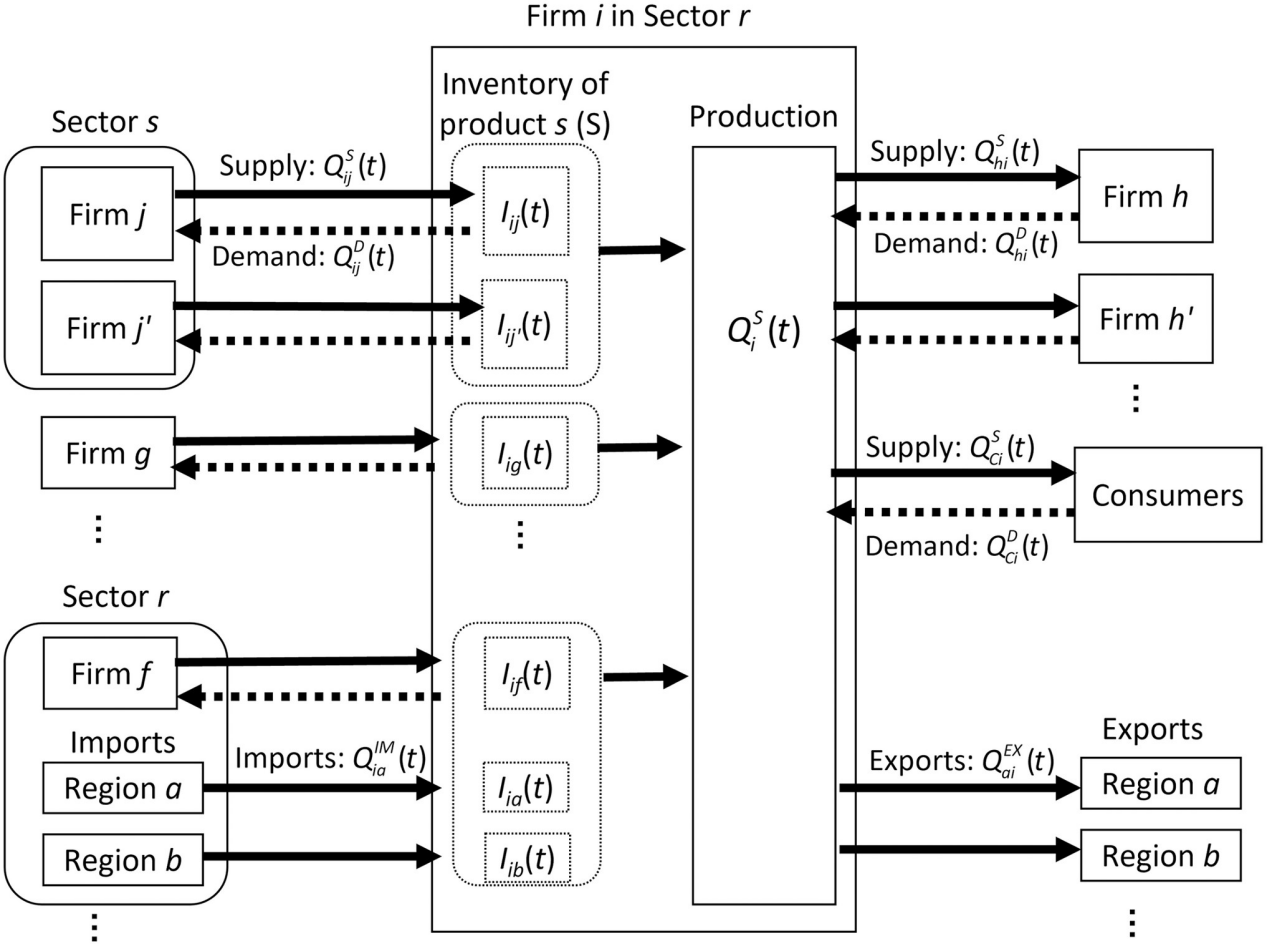
Overview of the agent-based model. Products flow from left to right, whereas orders flow in the opposite direction.

We extend dynamic agent-based models that focus on domestic supply chains by incorporating the imports of inputs and exports of outputs [21–23, 52]. The first part of this subsection and Fig 1 provide an overview of the model, with further details provided in the latter.

The model assumes that firms in a country are linked not only with each other through domestic supply chains but also with foreign input and output markets through international trade, as illustrated by the arrows in Fig 1. Each firm utilizes a fixed amount of labor and various intermediates provided by its domestic suppliers (for example, $Q_{ij}^{S}$ in the figure) and imported from foreign countries ($Q_{ia}^{\text{IM}}$), produces its product, and sells it to domestic ($Q_{hi}^{S}$) and foreign client firms ($Q_{ai}^{\text{EX}}$) and final consumers ($Q_{Ci}^{S}$).

Following a Leontief production function, each firm utilizes a certain amount of each intermediate good and labor to produce one unit of product. Which and how many intermediate goods are required vary across firms and are determined by the data. Products are sector specific, and hence, all firms in a particular sector produce the same product. Sectors are defined by the Japan Standard Industrial Classification in 2013 [53] and categorized into 1,460 classifications. Because our data include values of imported inputs from each broadly defined foreign region but not the product type of such imported inputs, we assume that the sectoral classification of the input imported by a firm is the same as that of the product of the firm.

Suppliers and clients are predetermined by the data and do not change in principle. In other words, even after the disruption of supply chains, firms cannot find any new supplier or client. However, in exercises in the Results and Discussion sections, firms are shown to be able to find other suppliers to identify product substitutions.

Each firm holds an inventory of intermediates purchased from each manufacturing firm in case of a shortage of supplies ($I_{ij}$), whereas no inventory is assumed for service inputs. Moreover, when the inventory of intermediates is from suppliers in the same sector, for example, firms *j* and *j*′ in Fig 1, they are considered substitutable. In addition, firms hold no inventory of their own product and immediately deliver it to clients and consumers.

We do not assume profit maximization, following other agent-based models for simplicity. Instead, we assume that each firm follows several rules that determine the demand for each intermediate good and the supply of its product to clients and consumers. In the initial period, or day 0 without any economic shock, the demand and supply of each firm’s product are the same. On day 1, an economic shock, such as a new policy or natural disaster, disrupts imports from particular foreign regions ($Q_{ia}^{\text{IM}}$). After the shock, firms directly facing disrupted supply chains (firms *h* and *h*′) reduce their production level due to the supply shortages of inputs. Conversely, the disruption of exports to certain regions ($Q_{ai}^{\text{EX}}$) affects exporters’ clients (firms *j* and *j*′). Furthermore, the shock propagates downstream and upstream to other firms through supply chains. Because of the possible reduction in the production level, the demand for a firm’s product may exceed its supply. If so, then the firm determines how its production is allocated to its client firms and consumers following a rationing rule.

#### Supply and demand

In the following, we denote the daily supply of the intermediate product of supplier *i* to client *h* on day *t* by $Q_{hi}^{S}\left(t\right)$, the supply to final consumers by $Q_{Ci}^{S}\left(t\right)$, and the exports to region *a* by $Q_{ai}^{\text{EX}}\left(t\right)$. Then, the production of firm *i* on day 0 is given by

$$\begin{array}{c} Q_{i}^{S}\left(0\right)=\Sigma_{h}Q_{hi}^{S}\left(0\right)+Q_{Ci}^{S}\left(0\right)+\Sigma_{a}Q_{ai}^{\text{EX}}\left(0\right). \end{array}$$ (1)

We assume that each firm predicts that the demand for its product on day *t* is equal to that on the previous day, $Q_{i}^{D}\left(t-1\right)$. To meet this demand, firm *i* needs supplier *j*’s product in a certain amount, $Q_{ij}^{S}\left(0\right)Q_{i}^{D}\left(t-1\right)/Q_{i}^{S}\left(0\right)$, because $Q_{ij}^{S}\left(0\right)$ represents the supply of supplier *j*’s product to firm *i* in the initial state, and $Q_{i}^{D}\left(t-1\right)/Q_{i}^{S}\left(0\right)$ is the ratio of current demand to initial supply.

In addition, firms demand intermediates to hold their inventories in case of supply chain disruption. Specifically, firm *i* has an inventory of the intermediate produced by firm *j* on day *t*, *I*_*ij*_(*t*), and intends to restore this inventory to a level equal to a given number of days, *n*_*i*_, of the utilization of supplier *j*’s product, $n_{i}Q_{ij}^{S}\left(0\right)$. We assume that *n*_*i*_ is randomly determined by a Poisson distribution, where the mean is *n* in the beginning of the simulation. Note that there are data indicating the inventory of each firm’s own products but not that of the intermediate goods used by each firm. Therefore, we rely on this probabilistic assignment. Following Inoue and Todo [22], *n* is set to 9 such that the model replicates the economic reaction to the Great East Japan earthquake. In addition, to avoid a bullwhip effect, i.e., large fluctuations across simulations, *n*_*i*_ is assumed to be greater than or equal to 4. When actual inventory *I*_*ij*_(*t*) is smaller than its target $n_{i}Q_{ij}^{S}\left(0\right)$, firm *i* increases its inventory gradually by 1/*τ* of the gap in one day such that it reaches the target in *τ* days. We assume that *τ* = 6, following an existing study [54].

Combined with the abovementioned two purposes, i.e., production and inventory, firm *i*’s demand for the product of its supplier *j* on day *t*, denoted by $Q_{ij}^{D}\left(t\right)$, is given by

$$\begin{array}{c} Q_{ij}^{D}\left(t\right)=Q_{ij}^{S}\left(0\right)\frac{Q_{i}^{D}\left(t-1\right)}{Q_{i}^{S}\left(0\right)}+\frac{1}{\tau }[n_{i}Q_{ij}^{S}\left(0\right)-I_{ij}\left(t\right)]. \end{array}$$ (2)

Inventory should not be considered for a service supplier. This aspect is realized by the second term and is thus omitted, and $Q_{ij}^{D}\left(t\right)$ is always equal to $Q_{ij}^{S}\left(0\right)$, where *j* belongs to the service sector.

Accordingly, total demand for the product of supplier *i* on day *t*, $Q_{i}^{D}\left(t\right)$, is given by the sum of total demand from its client firms and final consumers and exports:

$$\begin{array}{c} Q_{i}^{D}\left(t\right)=\Sigma_{h}Q_{hi}^{D}\left(t\right)+Q_{Ci}^{D}+\Sigma_{a}Q_{ai}^{\text{EX}}\left(0\right). \end{array}$$ (3)

On day 0, we assume that the level of inventory is equal to its target level ($n_{i}Q_{ij}^{S}\left(0\right)=I_{ij}\left(0\right)$) and that the demand for the product of firm *i* on the previous day is equal to its production ($Q_{i}^{D}\left(t-1\right)=Q_{i}^{S}\left(0\right)$). Therefore, there is no excess supply or demand on day 0: $Q_{ij}^{S}\left(0\right)=Q_{ij}^{D}\left(0\right)$ and $Q_{i}^{S}\left(0\right)=Q_{i}^{D}\left(0\right)$.

#### Disruption of supply chains

Now, let us suppose that the imports of intermediate goods from some foreign countries or regions are disrupted because of a new trade policy or natural disaster. When facing a shortage of imports of an intermediate product from region *a*, firm *i* can use its inventory of the intermediate good, including the inventory of the same intermediate good from other domestic suppliers in the same sector and imports from other regions. Therefore, the maximum possible production level of firm *i*, limited by the product inventory of sector’s intermediate good *s* on day *t*, ${\bar{Q}}_{i(s)}^{S}\left(t\right)$, is given by

$$\begin{array}{c} {\bar{Q}}_{i(s)}^{S}\left(t\right)=\frac{\Sigma_{j\in s}I_{ij}\left(t\right)}{\Sigma_{j\in s}Q_{ij}^{S}\left(0\right)}Q_{i}^{S}\left(0\right), \end{array}$$ (4)

where Σ_*j*∈*s*_*I*_*ij*_(*t*) is firm *i*’s total inventory of the intermediate of sector *s*, including imports, on day *t* and $\Sigma_{j\in s}Q_{ij}^{S}\left(0\right)$ is the amount of intermediate good *s* required to produce the initial production level of firm *i*. Because we assume a Leontief production function, the maximum possible production level of firm *i* on day *t* is constrained by the availability of inputs and given by the following:

$$\begin{array}{c} Q_{\text{max}i}^{S}\left(t\right)={\text{Min}}_{s}\left({\bar{Q}}_{i(s)}^{S}\left(t\right)\right). \end{array}$$ (5)

Therefore, the supply of firm *i* on day *t* is determined either by the maximum production capacity when it is smaller than demand or by demand and thus given by

$$\begin{array}{c} Q_{i}^{S}\left(t\right)=\text{Min}(Q_{\text{max}i}^{S}\left(t\right),Q_{i}^{D}\left(t\right)). \end{array}$$ (6)

In alternative scenarios, we assume that the exports of products to some foreign countries or regions are stopped due to a new trade policy or natural disaster. In this case, the demand for firms exporting to foreign regions declines by the amount of exports. The supply of firm *i* is still determined by Eq (6).

In either scenario, the supply and demand of firms that are not directly engaged in international trade may be affected by supply chain disruption because of the propagation of its effect throughout supply chains.

#### Rationing of production

When the total demand for firm *i*’s product is greater than its production capacity, the firm cannot satisfy the demand of its clients and consumers and thus has to ration its production to them. Suppose that firm *i* has clients *h* ∈ {1, …, *H*}, final consumers, and importers. The supply to each client, consumer, and importer is determined by the following steps, where the demand of agents, which is relatively small compared with their initial demand, is prioritized [22]. To explain the associated procedure, let us define the amount of production that has not been rationed and remains to be rationed at the beginning of step *x* as $Q_{i}^{R}\left[x\right]$. We also denote the minimum ratio of current demand to initial demand by $q_{\text{min}}^{D}\left(t\right)\equiv \text{Min}\left(q_{hi}^{D}\left(t\right),q_{CEXi}^{D}\left(t\right)\right)$. Here, $q_{hi}^{D}\left(t\right)\equiv Q_{hi}^{D}\left(t\right)/Q_{hi}^{S}\left(0\right)$ is the ratio of the demand of client *h* for the product of firm *i* to its initial demand and $q_{CEXi}^{D}\left(t\right)\equiv \left(Q_{Ci}^{D}\left(t\right)+\Sigma_{a}Q_{ai}^{\text{EX}}\left(t\right)\right)/\left(Q_{Ci}^{S}\left(0\right)+\Sigma_{a}Q_{ai}^{\text{EX}}\left(0\right)\right)$ is the corresponding ratio of the sum of the demand of final consumers and importers.

In the first step, *x* = 1 and $Q_{i}^{R}\left[1\right]=Q_{i}^{S}\left(t\right)$ by definition. At every step, the following equation is evaluated:

$$\begin{array}{c} Q_{i}^{R}\left[x\right]\geq q_{\text{min}}^{D}\left(t\right)Q_{i}^{D}\left(t\right). \end{array}$$ (7)

If Eq (7) holds, then firm *i* rations to each client firm, consumer, and importer the amount of its demand multiplied by the minimum demand ratio $q_{\text{min}}^{D}\left(t\right)$. The remainder of the production, $Q_{i}^{R}\left[x+1\right]=Q^{R}\left[x\right]-q_{\text{min}}^{D}\left(t\right)Q_{i}^{D}\left(t\right)$, flows to the next step. In addition, the demand from each client firm, consumer, and importer is removed by the ratio *q*^D^. Therefore, a client firm or those aggregate consumers and importers that satisfy its demand (or whose ratio of current demand to initial demand is the minimum) is dropped. In contrast, if Eq (7) does not hold at some step *x*, then firm *i* rations to each client, consumer, and importer the amount of its demand multiplied by the ratio of the remaining production to demand defined by $q_{\text{r-d}i}^{D}\equiv Q_{i}^{R}\left[x\right]/Q_{i}^{D}\left(t\right)$. At this step, the procedure ends because $Q_{i}^{R}\left[x+1\right]$ becomes zero.

Under this rationing policy, the inventory of firm *j*’s product held by firm *i* on day *t* + 1 is updated to

$$\begin{array}{c} I_{ij}\left(t+1\right)=I_{ij}\left(t\right)+Q_{ij}^{S}\left(t\right)-Q_{ij}^{S}\left(0\right)\frac{Q_{i}^{S}\left(t-1\right)}{Q_{i}^{S}\left(0\right)}. \end{array}$$ (8)

This equation, combined with Eqs (2) and (6), determines the demand of firm *i* for the intermediate good supplied by firm *j* on day *t* + 1, $Q_{ij}^{D}\left(t+1\right)$, and the total demand for firm *i*’s product, $Q_{i}^{D}\left(t+1\right)$. The supply of firm *i* on day *t* + 1, $Q_{i}^{S}\left(t+1\right)$, is then determined by Eq (6).

### Simulations

Using the agent-based model and firm-level data with the supply chain information explained above, we simulate how the disruption of imports or exports due to a new trade policy or natural disaster affects the total production level in Japan. In particular, we simulate the model using numerous scenarios considering five dimensions: (1) the type of trade (import, export, or both), (2) the target region (the world, Asia, China, Asia except for China, North America, Europe, the Middle East, and others), (3) the duration of disruption (two, four, or six weeks or two months), (4) the strength of disruption, i.e., the rate of reduction in imports from or exports to the target region (20, 40, 60, or 80%), and (5) those industries whose imports or exports are disrupted (all or one of the manufacturing industries).

For example, in one scenario, we assume a reduction in the imports of all intermediate products from the world by 60% for four weeks and simulate the total production level of Japan day by day. In another scenario, we assume a reduction in the amount of imports of electric machinery, equipment, and supplies from China by 80% for two months.

We run numerous simulations under these different scenarios. Then, we calculate the total reduction and the ratio of the reduction in total production due to the disruption to the total production without any disruption during the disruption period, denoted as the reduction rate.

**Fig 2:**
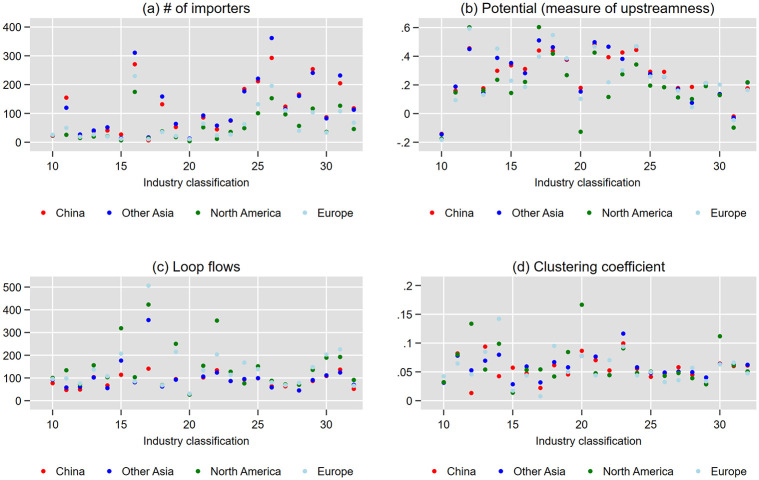
Average network topology of importing firms by industry and region. The horizontal axis indicates the Japan Standard Industrial Classification (JSIC) two-digit industries of importers, whereas the vertical axis indicates the mean of the number of importing firms and their average potential, loop flows, and clustering coefficients. The color of each dot represents the exporting country.

We start with national-level simulations where imports or exports of any industry are assumed to be disrupted, but then, we focus on industry-level simulations assuming the disruption of imports or exports of a particular industry for the following four reasons. First, in practice, trade restrictions due to national security concerns often target limited industries, particularly high-tech industries [11]. Second, supply chain resilience and vulnerability are found to vary across industries [55]. Third, as we see later in Fig 2, the average network topology of importers varies substantially across industries. Finally, how the effect of industry-specific disruption spills over across industries may vary across industries [4].

The simulations in this paper are different from those in previous studies that examine the effect of natural disasters [21–23, 52]. Notably, while the previous simulations assume supply chain disruptions due to damage to domestic production facilities by disasters and gradual recovery from the damage, our simulations assume reductions in trade for a particular period and immediate recovery afterward.

### Helmholtz–-Hodge decomposition

HHD decomposes a flow in a network into a potential flow component and a loop flow component. A potential flow from one node to another is determined by the degree of upstreamness/downstreamness of the nodes in a network [42], whereas loop flows are given by the constraint that the summation of the incoming and outgoing loop flows of any node equals zero. This method can be applied to any network to compute potential and loop flows, even if the network is complex [43, 56–58].

Specifically, HHD is explained by the following equations. Suppose that a network is denoted by a set of flows from node *i* to *j*, represented by *X*_*ij*_. For simplicity, we assume that ∀*i*, *j X*_*ij*_ ≥ 0. *A*_*ij*_ is a binary adjacency matrix generated from *X*_*ij*_: *A*_*ij*_ equals 1 if *X*_*ij*_ > 0 and 0 otherwise. We define a “net flow”, *F*_*ij*_ by *F*_*ij*_ = *X*_*ij*_ − *X*_*ji*_, and a “net weight”, *w*_*ij*_ by *w*_*ij*_ = *A*_*ij*_ + *A*_*ji*_.

Note that *w*_*ij*_ is symmetric and nonnegative, that *w*_*ij*_ = *w*_*ji*_, and that *w*_*ij*_ ≥ 0 for any pair of *i* and *j*.

Then, the HHD value is given by

$$\begin{array}{c} F_{ij}=F_{ij}^{\left(c\right)}+F_{ij}^{\left(p\right)}, \end{array}$$ (9)

where loop flow $F_{ij}^{\left(c\right)}$ satisfies

$$\begin{array}{c} \sum_{j}F_{ij}^{\left(c\right)}=0, \end{array}$$ (10)

meaning that the loop flows are divergence free. Potential flow $F_{ij}^{\left(p\right)}$ can be expressed as

$$\begin{array}{c} F_{ij}^{\left(p\right)}=w_{ij}\left(\phi_{i}-\phi_{j}\right), \end{array}$$ (11)

where *ϕ*_*i*_ is the potential of node *i* that identifies its degree of upstreamness in the network. Eq (11) indicates that potential flow $F_{ij}^{\left(p\right)}$ is the difference in the potential between two nodes when they are linked and zero when they are not linked. We further assume that

$$\begin{array}{c} \sum_{i}\phi_{i}=0 \end{array}$$ (12)

for normalization purposes. Then, Eqs (9)–(12) can be uniquely solved for $F_{ij}^{\left(c\right)}$, $F_{ij}^{\left(p\right)}$, and *ϕ*_*i*_ for all *i* and *j* in the whole network.

Fig 5 in S1 Appendix shows the average potential of importers from various regions by industry, indicating that manufacturing industries (codes 9–32) are relatively upstream, while wholesale (50–55), retail (56–61), and finance (62–67) industries are more downstream, confirming the finding in the literature [43].

### Regressions

Using the simulation results explained above, we run regressions to quantify the effect of the network topology of importers in a particular manufacturing industry importing from a particular region on the level of production loss due to the disruption of the imports of importers for a particular period. In other words, our analysis is at the industry-region level rather than at the firm level, assuming the disruption of the imports of each importer firm. The reason for this is that trade disruption does not usually occur at the firm level but rather at a more aggregate level, such as the industry or country level.

We first hypothesize that the number of importers and their centrality in domestic supply chains may positively affect production loss, as the two are related to the number of nodes affected initially and subsequently. To measure the centrality of importers, we use the average of their degree and betweenness centrality.

Second, we hypothesize that if importers are located in more upstream positions, i.e., if they import less assembled material, parts, or components, then the effect of import disruption can affect more firms through long supply chains to the bottom and thus result in a larger loss. To compute the degree of upstreamness of each firm, we employ HHD [42]. Using HHD, we can decompose a flow from one node to another into potential (hierarchical) and loop (horizontal) flows and thus compute the “potential” of each node that can be regarded as a measure of its degree of upstreamness [41, 43]. Our firm-level measure of the degree of upstreamness can capture heterogeneity in the degree of upstreamness across firms within the same industry, unlike the industry-level measures of the degree of upstreamness often used in the literature [59–63]. We take the average of the potential of importers in each industry from each region and use it as a measure of the degree of upstreamness of importers.

Finally, we examine how loops affect propagation through supply chains. Supply chains consist of numerous loops in a complex way [22, 41] because upstream suppliers may use final products, such as machinery and computers, produced by downstream assemblers. If an economic shock from import disruption is confined in a loop and does not affect firms outside the loop, then the production loss can be smaller than it would be otherwise. Therefore, we hypothesize that the number of loops that pass through importers is negatively correlated with production loss. Using HHD, we can compute the number of loop flows in which a node is involved in a network. In addition, we utilize a more widely used measure, the local clustering coefficient of a node, which is defined as the ratio of the number of actual links between nodes linked with the focal node to the number of all possible links between them. We use importers’ average of the number of loops and the clustering coefficient.

We test these hypotheses with ordinary least squares (OLS) estimations of the following equation:

$$\begin{array}{c} \text{ln}\;{ProdLoss}_{sc}=\alpha +\beta \;\text{ln}\;{DisruptImp}_{sc}+X_{sc}\delta^{\prime }+\epsilon_{sc}, \end{array}$$ (13)

where ${ProdLoss}_{sc}$ indicates the predicted total loss in value added production in Japan due to the disruption of imports from country *c* to industry *s* by 80% for 28, 42, or 60 days and ${DisruptImp}_{sc}$ is the value of disrupted imports. The value of disrupted imports is always controlled for in the regressions because Fig 6 shows its correlation with production loss. *X*_*sc*_ indicates a vector of variables that represent the network topology of importers in industry *s* from country *c*, namely, the number of importers, potential, loop flows, and local clustering coefficient. The distribution of these variables is shown in Fig 6 of S1 Appendix. We run separate regressions using data at the exporter country-industry level from simulations assuming different durations of import disruption to examine how the effect of network topology differs depending on the duration. In the benchmark specification, we include all the variables for network topology in each regression and illustrate the results in Fig 7. In addition, we experiment with those specifications where only one of the topology variables is used as *X*_*sc*_ in Eq (13) and show the results in Tables 4–6 of S1 Appendix, confirming the robustness of the benchmark results.

### Network topology

The supply chains in the sample show several notable characteristics as a network. First, the median number of links of each firm, or the degree, is four, and the mean is 9.07, whereas there are two firms whose degree is more than 10,000 and 19 firms whose degree is more than 5,000. This finding means that the degree distribution follows a power-law distribution [22], as is commonly observed in many natural and social networks [30]. Second, the average number of steps between firms, or the average path length in supply chains, is 4.8, resulting in a small-world network [64]. Third, 46–48% of the firms are included in the giant strongly connected component (GSCC), in which all firms are directly or indirectly connected through directed links, as found in previous studies using the same data [22, 65]. These network characteristics would magnify propagation through supply chains [22].

**Fig 3:**
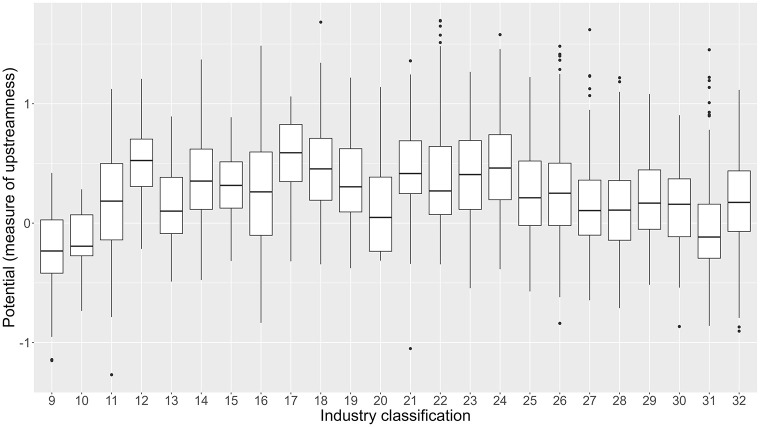
Distribution of the potential of importing firms by industry. The horizontal axis indicates the Japan Standard Industrial Classification (JSIC) two-digit industries of importers, whereas the vertical axis indicates the mean of the potential of importing firms. The color of each dot represents the exporting country.

The average of the number of importer firms, their measures of potential and loop flows, and their clustering coefficient by industry and exporter region is shown in Fig 2. The four panels of the figure show that the average network topology of importers varies substantially across industries and exporter regions. For example, the potential is higher for the rubber (code 12), petroleum (17), plastic (18), ceramic (21), iron (22), nonferrous metal (23), and metal product (24) industries and lower for the transportation equipment industry (31). The potential of the general, production, and business machinery (25–27), electronic parts (28), electric machinery (29), and information and communication equipment (30) industries is in the middle. Therefore, it is worth assuming industry-specific import disruption in the simulations. In addition, Fig 3 exhibits a large variation in potential across firms in each industry. For example, the potential of some firms in the transportation equipment industry is quite high, although its industry average is low. Although this finding suggests that simulations assuming firm-specific trade disruption may also be interesting, we stick with national-level and industry-specific simulations because, in practice, trade disruption often occurs at the national or industry level and not at the firm level.

## Results

### National-level import disruption

We estimate the value of the loss in total value added production in Japan due to the disruption of the imports of inputs from the world or from various regions by 20–80% for a duration from two weeks to two months. Throughout the main paper, we focus on the disruption of imports because the effect of import disruption is found to be substantially larger than is the effect of export disruption. Results in S1 Appendix presents the results from the disruption of exports to various regions and the disruption of imports and exports.

**Fig 4:**
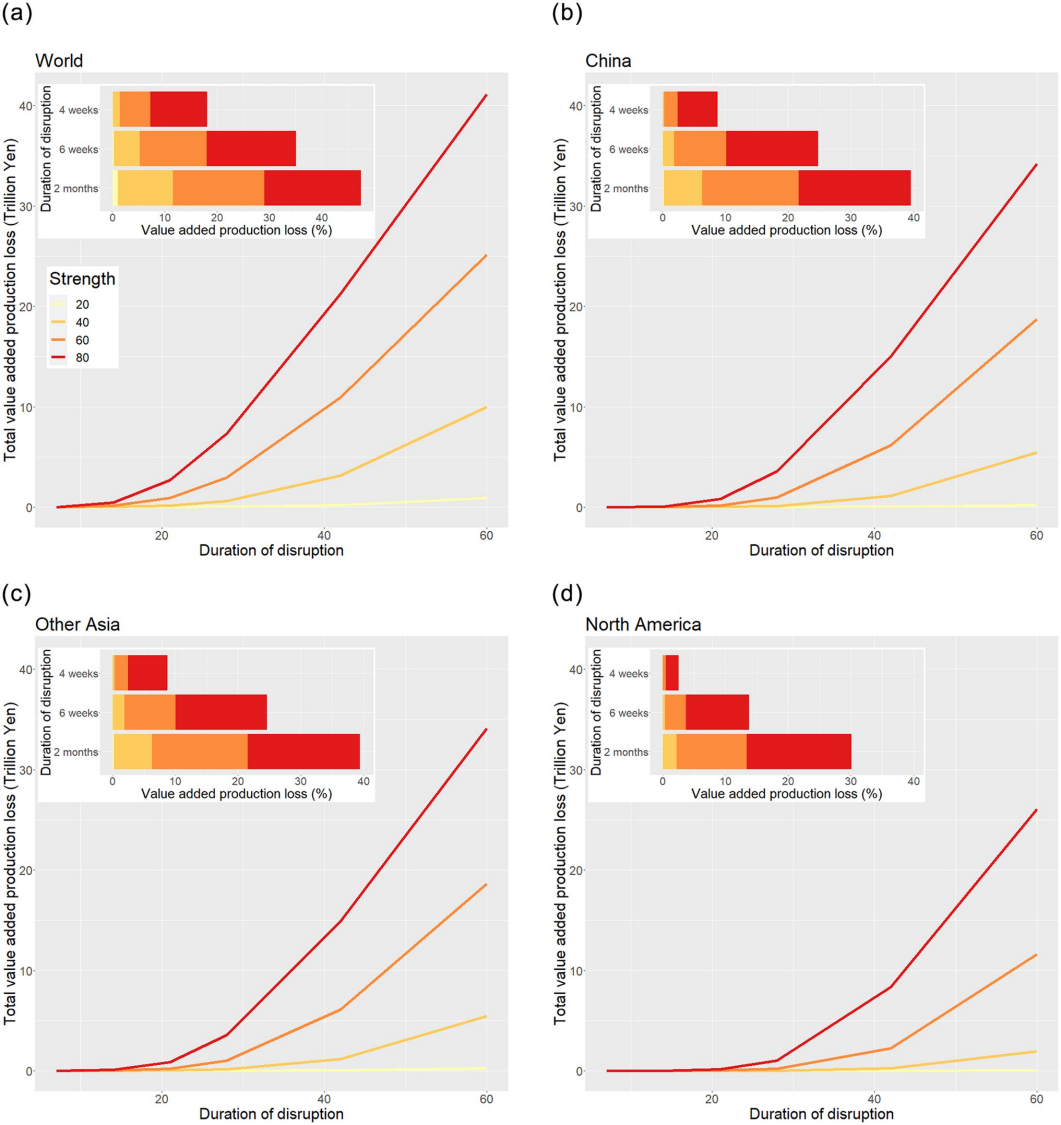
Effect of the disruption of imports from various areas on domestic production. The main panels in the figure show the total loss in value added production in Japan when imports from each region are disrupted at a particular strength (20, 40, 60, or 80%) for a particular duration (from 2 weeks to 2 months). The inset panels show the ratio of the loss in value added production to total production when the imports from each region are disrupted at a particular strength for 4 and 6 weeks and 2 months. The right edge of each color of the bars indicates the loss rate at the corresponding strength. “Other Asia” represents Asian countries except for China.

Fig 4 depicts the relationship between the duration of import disruption at a particular strength (a reduction in imports by 20–80%) and the loss in value added production due to this disruption, whereas the inset in the same figure illustrates the relationship between the duration and ratio of loss to total value added production without disruption in the same periods. The three findings presented below should be emphasized.

First, the effect of import disruption is substantially magnified by propagation through domestic supply chains. When imports from the world decrease by 80% for two months (60 days), the total value of disrupted imports is 5.9 trillion yen, whereas the reduction in total value added production due to this disruption is 41.1 trillion yen, or approximately 7 times as large as that for disrupted imports (upper-left panel in Fig 4). The loss in value added due to import disruption accounts for 47.5% of total value added production, as shown by the inset in this figure.

Second, the loss in value added production is not proportional to the duration of import disruption. Disruption at any strength for two weeks causes a negligible reduction in production because firms are assumed to hold inventories of intermediate goods for nine days of their use on average. However, the reduction rate becomes nonnegligible four weeks after the start of the disruption when its strength is high: 7.2 and 18.1% due to a reduction in imports from the world by 60 and 80%, respectively (inset of the upper-left panel in Fig 4). When the disruption lasts for two months, i.e., an approximately doubled duration, the corresponding reduction rate becomes far larger: 29.0 (4.0 times) and 47.5% (2.6 times). This exponential growth of the reduction in production is due to the propagation of the effect of the disruption of imports to the direct and indirect clients of importers through supply chains.

Finally, as the strength of disruption increases, the loss in value added production also increases exponentially, rather than proportionally. When the level of a 2-month disruption doubles from 40 to 80%, production loss becomes 4 times as large, from 10.0 trillion yen (11.5% of the total value added for the 2 months) to 41.1 trillion yen (47.5%) (upper-left panel in Fig 4). These results suggest that a large initial shock is more likely to result in cascading through supply chains and to be magnified than is a small shock.

Fig 4 also depicts the simulation results assuming the disruption of imports from various regions that are defined by our data: China, Asia except for China (other Asia), and North America. Our data also include Europe, the Middle East, and other areas as partner regions, but the results are not shown here for simplicity (see Results in S1 Appendix). The results in all panels share the same 3 characteristics as the abovementioned simulation of the disruption of imports from the world. Comparing the panels, we observe that the largest effect comes from the disruption of imports from China, followed by those from other Asia. The disruption of imports from China and other Asia for two months by 80% reduces total value added production by 39.5 and 39.4%, respectively. Although the amount of disrupted imports from China, 1.1 trillion yen in our data, is much smaller than that from other Asia, 1.8 trillion yen, the associated reductions in value added production are approximately the same. This finding highlights that the effect of the disruption of imports from China is particularly magnified through supply chains.

### Industry-specific import disruption

We further investigate how a reduction in imports in a particular manufacturing industry by 80% for six weeks affects production in other industries. Those industries for which imports are disrupted are limited to manufacturing industries and defined at the two-digit level according to the Japan Standard Industrial Classification [53]. Those industries that are affected by such disruption are defined at the one-digit level for nonmanufacturing industries and at the two-digit level for manufacturing industries (Tables 1 and 2 in S1 Appendix).

**Fig 5:**
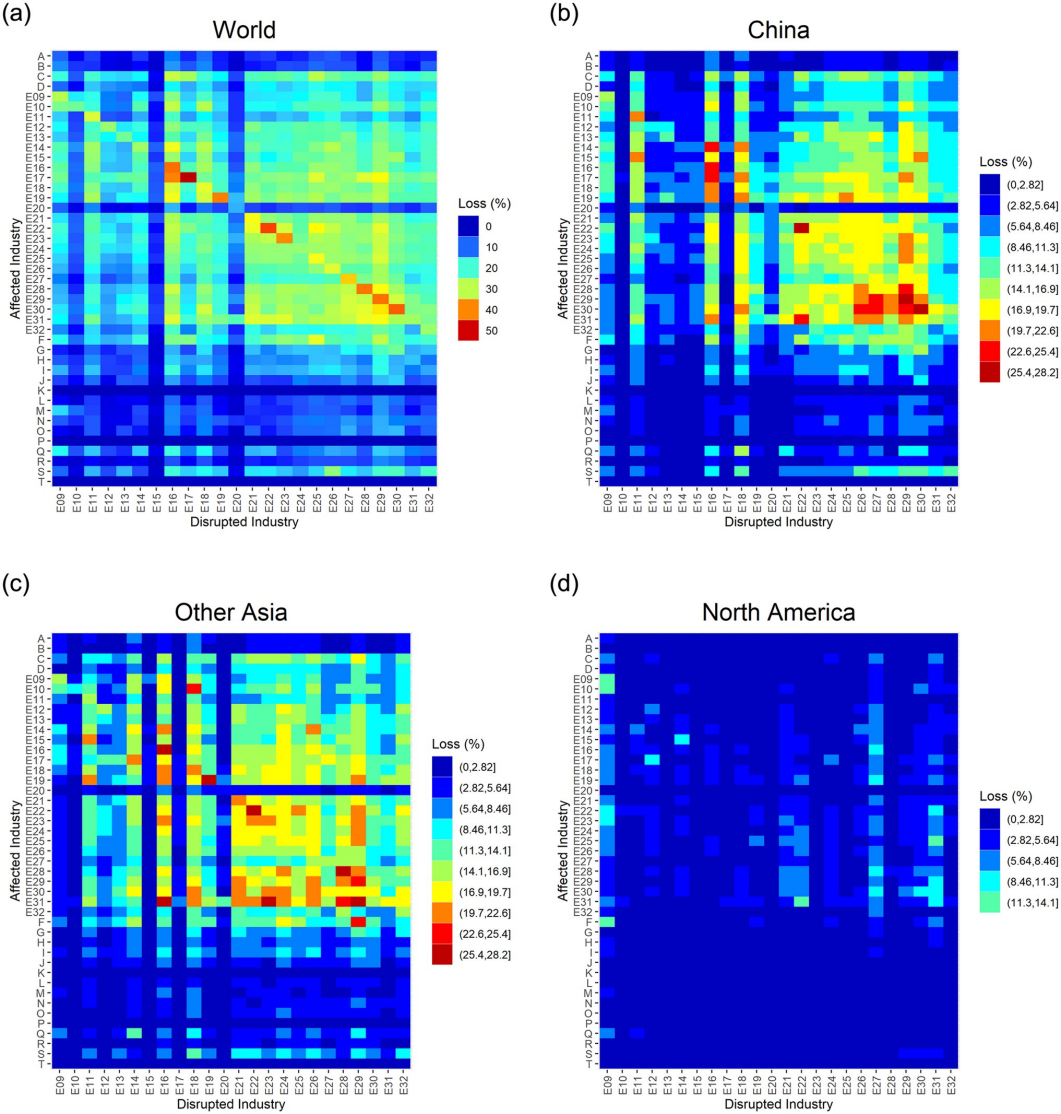
Effect of import disruption on production by industry and exporter region. The heatmaps show the ratio of loss in the value added production of each industry, on the vertical axis, to its total production when imports to a particular industry, on the horizontal axis, from a particular region, labeled at the top of each panel, are disrupted by 80% for 6 weeks. Those industries on the horizontal axis (disrupted industries) are at the two-digit level in the manufacturing sector, whereas those industries on the vertical axis (affected industries) are at the one-digit level for nonmanufacturing industries and at the two-digit level for manufacturing industries. The color separations in the panel labeled “World” are defined independently, whereas those in the other panels are commonly defined.

The results are presented in Fig 5. The upper-left panel shows the effect of the disruption of imports from the world to a particular industry (horizontal axis) on various industries (vertical axis). Several findings are notable. First, the disruption of manufacturing imports (industries E09-E32) substantially reduces production in most manufacturing sectors, while its effect on nonmanufacturing sectors (A-D and F-T), except for the mining industry (C), is limited. Second, among manufacturing industries (E), the disruption of imports in most light industries, such as the food and beverage (9–10), wood and furniture (12–13), paper (14), and leather (20) industries, does not largely affect other manufacturing industries. In contrast, when a heavy or high-tech manufacturing industry (15–32 except for 20) is affected, the effect propagates to other industries.

In the other panels of Fig 5, we focus on the disruption of imports from selected regions, i.e., China, other Asia, and North America. The comparison among the three panels clearly shows that the effect of the disruption of imports from China and other Asia to any industry is far greater than that from North America. In addition, the disruption of imports from China and other Asia results in similar patterns of interindustry propagation. Notably, interindustry propagation is more prominent when imports to industries, such as the chemical (16), plastic (18), production machinery (26), business machinery (27), electronics (28), electrical machinery and equipment (29), and information and communication electronics equipment (30) industries, are disrupted.

**Fig 6:**
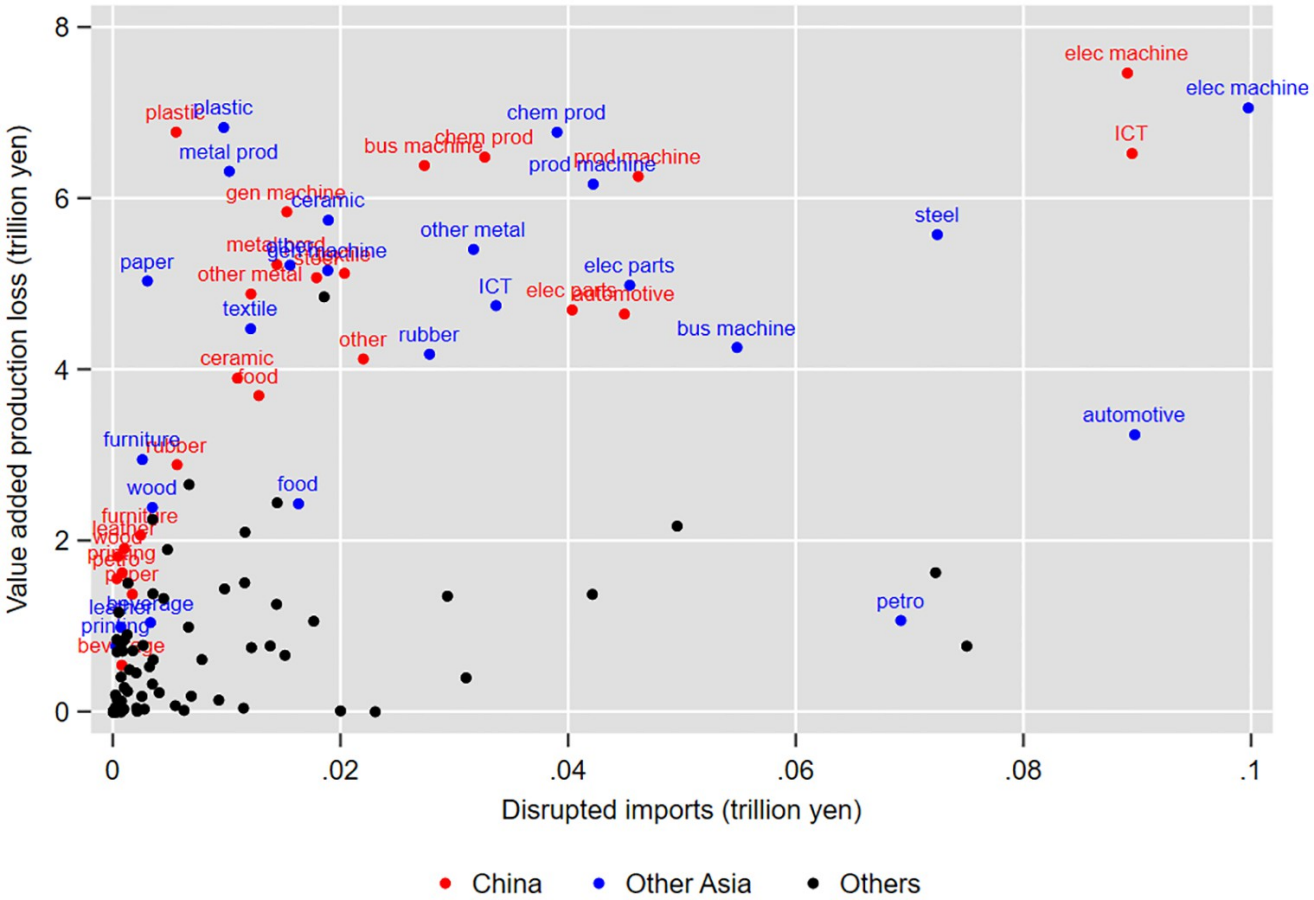
Relationship between disrupted imports and the resulting production loss by industry. This figure shows the correlation between a reduction in the amount of imports of a particular industry in the manufacturing sector from each region by 80% for 6 weeks and the reduction in the level of production due to import disruptions (both in trillions of yen). Red, blue, and black dots indicate imports from China, other Asia, and other regions except for Asia, respectively. The labels above the red and blue dots indicate their abbreviated industry names. The complete industry classifications are shown in detail in Table 2 of S1 Appendix.

Fig 6 further shows the relationship between the value of disrupted imports to a particular industry from each region and the production loss in the whole economy due to import disruption. In Fig 6, red, blue, and black dots represent import disruptions from China, other Asia, and all others, respectively. The labels above the red and blue dots indicate abbreviated industry names. We find that the top industries in terms of the effect of the disruption of imports from China and other Asia include the electrical machinery, ICT equipment, plastic, chemical, and general, business, and production machinery industries. These findings suggest that the disruption of imports, particularly from China and other Asia, to selected industries, most notably the electrical machinery and equipment industry, will reduce the level of production not only in these industries but also in the whole Japanese economy via the propagation of economic shocks through supply chains.

Another notable finding from Fig 6 is that production loss is not necessarily proportional to the value of disrupted imports. For example, the disruption of imports from China or other Asia to the plastic and metal products industries would cause large production loss, although their volumes of disrupted imports are small. Therefore, we now turn to how this production loss propagates through supply chains, focusing on the role of the network topology of importers.

### Effect of network topology on propagation

**Fig 7:**
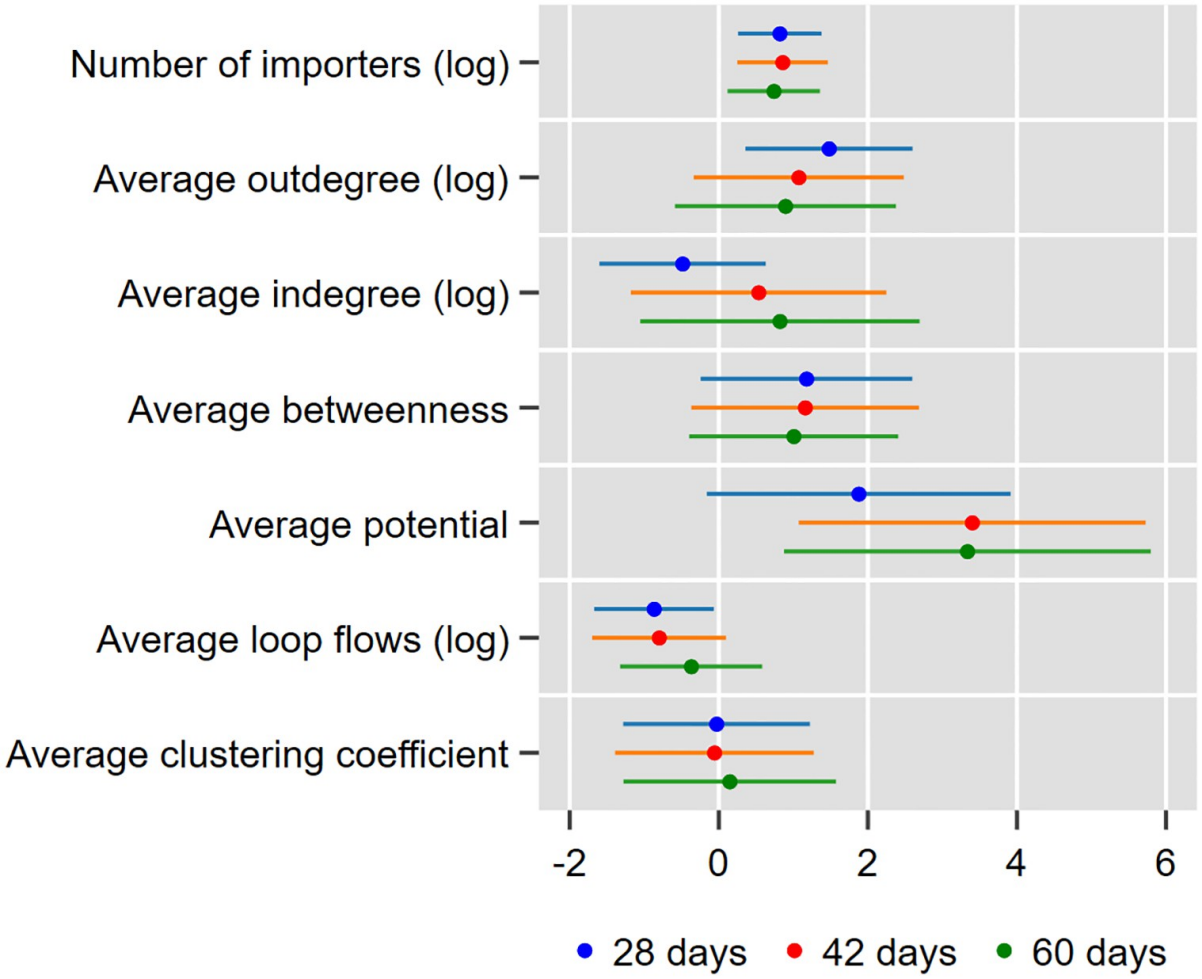
Effects of the network topology of importers on production loss. This figure shows the coefficients and their 95% confidence intervals from three regressions of the decline in the level of production in logs due to the disruption of imports from a particular region to a particular manufacturing industry for 4 weeks, 6 weeks, or 2 months. In each regression, we control for the value of disrupted imports in logs. To make the sizes of coefficients comparable across variables, we multiply the average clustering coefficient by 10 and average betweenness by 10,000.

We estimate the average network topology of importers using the framework explained in the regression subsection. The estimated coefficient of each variable and its 95% confidence interval are shown in Fig 7. Blue, orange, and green dots and lines indicate the results from the disruption of imports for 4 weeks, 6 weeks, and 2 months, respectively. The full results and those from alternative specifications are shown in Tables 4–6 of S1 Appendix.

We find that when imports from a region are disrupted, the number of importers has a positive and significant effect on the loss of value added production due to import disruption, regardless of the duration of such disruption. The values of the significant coefficients imply that an increase in the number of importers by one standard deviation is associated with a substantial increase in production loss by 80–94%.

However, the effect of some other variables varies depending on the duration of import disruption. The outdegree, or the number of clients of importers, positively affects production loss when the duration is 4 weeks; this effect is large because an increase in the outdegree by one standard deviation raises production loss by 70%. However, the effect of the outdegree is smaller and nonsignificant when the duration is longer than 4 weeks. Similarly, the measure of loop flows is negatively correlated with production loss, but the correlation is significant only when the duration is 4 weeks. In contrast, the effect of the potential, a measure of the degree of upstreamness, is nonsignificant when the duration is 4 weeks but becomes positive and significant when the duration is 6 weeks or 2 months. Furthermore, an increase in the potential by one standard deviation leads to an increase in production loss in 2 months by 62%.

These results imply that in the short run, the outdegree of importers and their involvement in loop flows substantially affects production loss, most likely because a higher outdegree results in immediate propagation to many firms and because the presence of loops helps confine the shock to these loops. However, in the long run, shocks propagate to more downstream indirect clients of importers, beyond their direct clients, and cannot be confined within loops of importers but rather escape outside. Therefore, the upstreamness of importers plays a more important role in propagation in the long run than do their outdegree and involvement in loops.

In addition, we find that the indegree, betweenness centrality, or clustering coefficient of importers has no significant effect using any duration once we control for the number of importers and thus use the average values of importers.

Furthermore, to examine possible heterogeneity in the effect of the centrality measures, we incorporate the interaction terms between the potential, outdegree and indegree in the regression analysis. We find that the effect of the outdegree is negative and nonsignificant, while the interaction between the potential and outdegree is positive and highly significant in all specifications, as shown in Table 1. This result indicates that although the effect of the average outdegree of importer firms, i.e., the average number of their clients, is mostly nonsignificant on average (Fig 7), its effect varies depending on the average degree of upstreamness of importers. Specifically, a higher number of clients of importers tends to result in larger production loss due to import disruption when importers are more upstream in supply chains but not necessarily when they are downstream. In other words, the effect of the disruption of imports to upstream importers with many clients propagates downstream widely and deeply through supply chains, affecting many firms in the economy.

**Table 1:** Regression results examining the heterogeneous effects of centrality. The dependent variable is the loss of value added production. Columns (1), (2), and (3) indicate the results from simulations assuming import disruption for 28 days, 42 days, and 60 days, respectively. All independent variables except for the reduction in the amount of imports are the average of importers.

VARIABLES	(1)	(2)	(3)
	28 days	42 days	60 days
Potential	0.668	3.808	5.431
	(4.960)	(5.865)	(6.822)
Outdegree (log)	-1.336	-2.311*	-2.181
	(0.835)	(1.364)	(1.561)
Outdegree (log) * Potential	9.487***	11.44***	10.06**
	(3.141)	(4.347)	(4.964)
Indegree (log)	2.368**	3.880**	3.900**
	(1.059)	(1.665)	(1.921)
Indegree (log) * Potential	-9.075**	-12.58**	-12.48**
	(3.645)	(5.173)	(6.002)
Reduction in the amount of imports (log)	0.584***	0.444***	0.262**
	(0.105)	(0.118)	(0.124)
Number of importers (log)	0.800***	0.866***	0.802***
	(0.281)	(0.272)	(0.295)
Betweenness	0.958	0.855	0.739
	(0.614)	(0.584)	(0.538)
Loop flows (log)	-0.889**	-0.847**	-0.383
	(0.381)	(0.385)	(0.415)
Clustering coefficient	-0.0916	-0.189	-0.0359
	(0.648)	(0.711)	(0.787)
Constant	-6.376**	-4.111	-3.345
	(2.667)	(2.604)	(2.850)
Observations	110	112	112
R-squared	0.692	0.636	0.502

Robust standard errors in parentheses

*** p<0.01,

** p<0.05,

* p<0.1

In contrast, the effect of the indegree, i.e., the number of suppliers of importers, is positive, whereas its interaction with the potential is negative (Table 1). Thus, the effect of the number of suppliers of importers is positive when importers are downstream but negative when they are upstream. Thus, the effect of import disruption propagates upstream widely and deeply when importers are downstream and linked with many suppliers.

These findings suggest that the standard measures of network topology often used in the literature, such as centrality or density, may not directly affect the long-run propagation of shocks through supply chains. However, the degree of upstreamness of affected nodes, which has not been well examined, plays a more important role, influencing the effect of these centrality measures. This finding implies that the key factors of propagation in supply chains are different from those in other standard networks because of the crucial role of the degree of upstreamness/downstreamness in supply chains.

### Substitution of suppliers

Given that the negative effect of import disruption is found to be magnified by domestic supply chains, the next question aims to address how propagation can be mitigated. We particularly examine the role of substitution among domestic suppliers suggested in the literature [6] by simulating three modified models assuming different levels of substitution.

**Fig 8:**
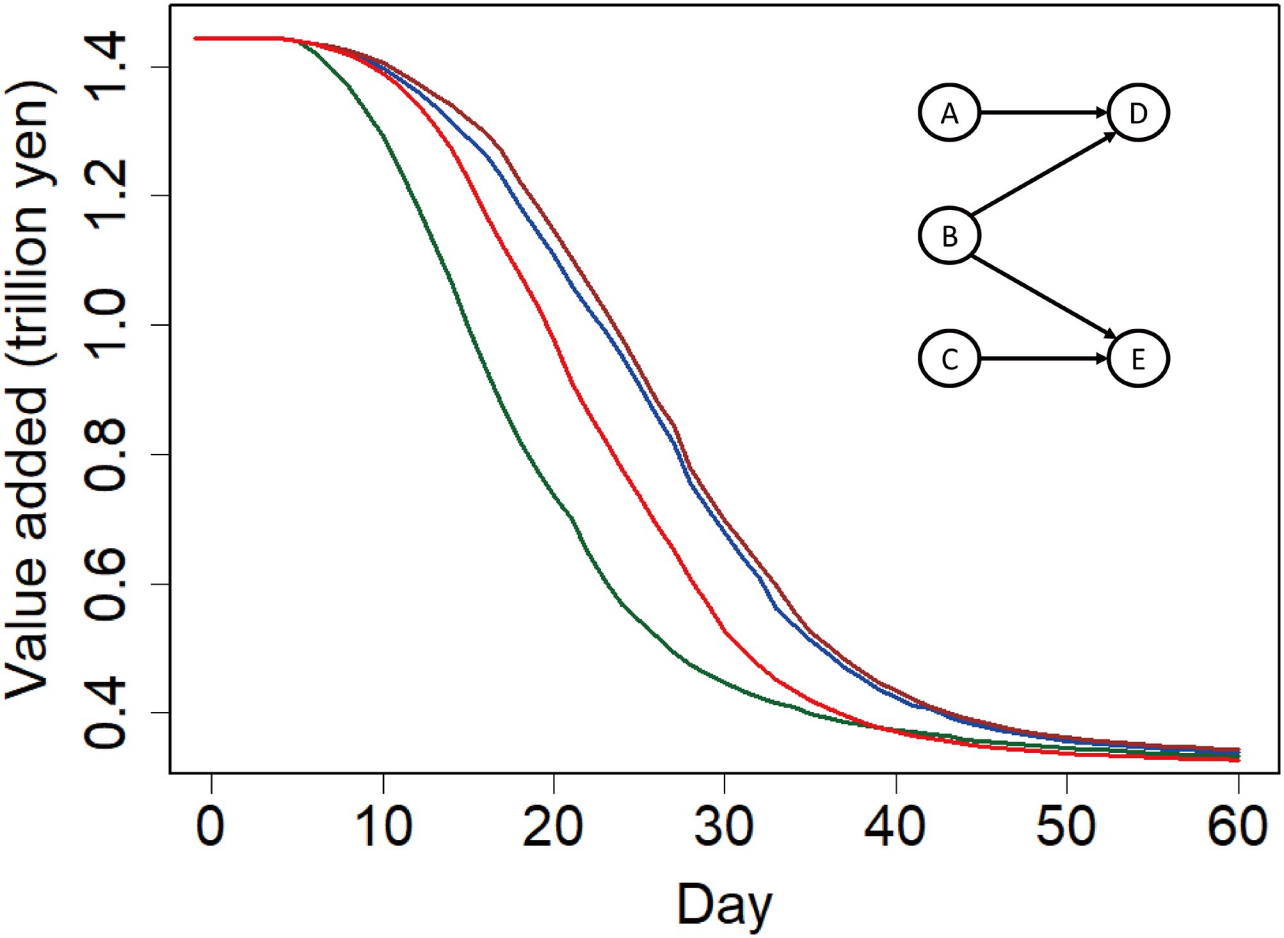
Decrease in production due to import disruption using different assumptions regarding supplier substitution. This figure illustrates the changes in daily total value added after the disruption of imports from the world by 80% for 60 days. Green, red, blue, and brown lines indicate changes assuming no supplier substitution, substitution between current suppliers in the same industry, substitution with new suppliers indirectly linked through supply chains, and perfect substitution with new suppliers, respectively. The inset panel illustrates how firms find new suppliers after supply chain disruption in the alternative model.

In our benchmark model, after import disruption, firms cannot be linked with suppliers or clients without any prior link to cope with supply chain disruption. However, in the first modified model, we assume that when a client firm faces a reduction in the transaction volume with one of its suppliers, it can be matched with any other supplier in Japan and procure disrupted supplies from the new supplier as long as the new supplier has the appropriate production capacity. Because this matching assumption may be too strong from a practical perspective, our second modified model alternatively assumes that a client firm facing a supply disruption can find another supplier not directly but indirectly linked through supply chains in two steps. The inset of Fig 8 indicates how the new supplier is found. Firm *D* is indirectly linked with supplier *C* through *B* and *E*. Let us suppose that *A* and *C* are in the same industry. Then, when the transactions between *A* and *D* decline after a shock, we assume that firm *D* can procure its supplies from *C*. This assumption that *D* can find *C* as its supplier is more plausible than is that in the first modified model that *D* can find a supplier from the entire network because *D* and *C* are indirectly connected and *A* and *C* are potential competitors. Such endogenous network shifts based on the current network are empirically found in the literature [66, 67]. Finally, to highlight the importance of supplier substitution, we also experiment with another model that assumes no substitution between suppliers, even when they are in the same industry.

Fig 8 shows the changes in daily value added production in Japan for 60 days predicted by the four models, assuming a disruption of 80% of imports from the world. Green, red, blue, and brown lines in the figure indicate the results assuming no substitutability of suppliers, substitutability between current suppliers (benchmark case), substitutability between network neighbors, and complete substitutability, respectively. Although the daily value added 2 months after the start of import disruption is similar across the 4 scenarios, it varies substantially up until 4 weeks. Accordingly, the cumulative value added production loss for the first 4 weeks is 13.5% of total value added without disruption under the substitutability of neighboring suppliers, while it is 18.1% in the benchmark case. Moreover, using alternative models assuming both no substitutability and complete substitutability, cumulative production is shown to decrease by 27.5 and 12.2%, respectively.

These results clearly suggest that the negative effect of import disruption can be mitigated by a more flexible reorganization of domestic supply chains. In addition, we find that matching with new suppliers indirectly linked through supply chains only a few steps away leads to a decline in production similar to that when assuming complete matching with any supplier. This finding implies that the reorganization of supply chains only within neighboring firms can substantially ameliorate the negative effect of import disruption.

## Discussion

Some of our findings are unique in the literature on network science. First, we find that the extent of the propagation of the effect of import disruption through the supply chain network is positively affected by the degree centrality of affected nodes (i.e., number of suppliers and clients of importer firms) in the short run. However, the average effect of the degree or betweenness centrality measure is nonsignificant in the long run once we control for the number of affected nodes. Our results contrast with other results showing that the centrality measures of nodes are major determinants of the diffusion of information and behavior from the nodes [38, 39, 68]. In contrast, our findings indicate that the degree of upstreamness of affected nodes is positively correlated with the long-run effect. Furthermore, although we find a nonsignificant average effect of degree centrality, it is heterogeneous and depends on the degree of upstreamness of affected nodes. To the authors’ best knowledge, these direct and indirect roles of the degree of upstreamness in diffusion have not been found in the literature.

Second, loops in supply chains are found to alleviate propagation in the short run because economic shocks can be confined to loops and may not extensively affect the economy outside the loops in the short run. This result contrasts with the results of some studies that clusters in a network can promote the diffusion of behavior, particularly health behavior, because such diffusion requires reinforcement from clustered neighbors [37, 40, 69]. One reason for the difference is that these studies focus on the “complex contagion” of behavior, rather than “simple contagion,” e.g., the infection of diseases that can be transmitted through a single contact between two persons. The propagation of economic shocks through supply chains is unlikely to be complex contagion, as defined by [40], because the disruption of the supply of one intermediate product used by a firm could completely stop its production. However, supply chain propagation is also different from simple contagion because of the possible use of inventory and the substitution of suppliers in the former. Our finding that the degree centrality of affected nodes does not necessarily magnify the total effect on the whole network is definitely different from what is usually observed under simple contagion.

Therefore, these two findings suggest that the propagation of economic shocks among firms through supply chains is quite different from the diffusion of information and behavior among individuals, highlighting the uniqueness of supply chains as a network.

Finally, our experiments reveal that substitution between only neighboring nodes indirectly linked a few steps apart in the network can mitigate the propagation of the negative effect as much as can substitution between distant nodes. Although the importance of supplier substitution for sustainable supply chains has been theoretically discussed [47, 50, 70] and empirically found [6, 22, 23, 31], such studies do not compare different types of substitution, i.e., neighboring versus random substitution, for higher resilience. Furthermore, although some studies in network science examine how the rewiring of nodes improves resilience in the context of airline and power grid networks [44, 46], they do not show that rewiring between network neighbors can effectively improve resilience.

Our findings also provide policy and managerial implications that are relevant to current circumstances where the risk of disruption of global supply chains is rising because of natural disasters and national security concerns.

First, we find that the effect of the disruption of the imports of upstream products on the importer economy is substantially magnified through domestic supply chains. Moreover, the effect of import disruption increases exponentially as its duration or strength increases. Therefore, policy makers should be careful about the large and growing negative effect when they impose trade restrictions to protect national security, minimizing the duration and coverage of these restrictions.

Second, our finding on the role of the network topology of importers implies that to minimize the economic effect of trade restrictions, policy makers should be concerned about the structure of supply chains, such as the number of trading firms and their degree of upstreamness, rather than simply focusing on the value of restricted trade.

Third, a reduction in the amount of imports for a short period would not induce a large production loss because firms hold inventories of intermediate goods. Therefore, one way to alleviate the negative effect of trade disruption is to increase the inventories of intermediate goods. The importance of inventory controls is often argued in the management literature [48, 50, 71], particularly during the COVID-19 pandemic [2].

Finally, firms are encouraged to plan for the possible substitution of neighboring suppliers in supply chains for affected suppliers in the event of supply chain disruption. One way to do so is to form strategic interfirm alliances, as suggested by [47, 48]. For this purpose, using digital technologies to collect and share information about product availability may be helpful. These strategies were utilized in practice during the COVID-19 pandemic and found to be effective [2, 72].

### Limitations and future works

Several caveats of this study should be noted. First, our benchmark model allows for changes in supply chain links after import or export disruption with predisruption partners but not with new partners. Second, our analysis is based on an agent-based model without any price. Because of these two shortcomings, our conclusions should be viewed with caution and applied only to short-term analysis. Therefore, the long-term scenarios where we assume import or export disruption for two months may lead to an overvaluation of the effect of supply chain disruption due to the rigidity of supply chains. Finally, our trade data are based on the BSJ data in which only large firms are included, and thus, imports are particularly undervalued (see the Data section). Because SMEs that import inputs often rely only on China, those imports from China in our data and their effect on production in our simulation may be underestimated.

Future works are expected to overcome these shortcomings using more realistic models and detailed data, such as transaction-level data used in recent studies [71, 73]. In addition, while this paper examines industry-specific trade disruption, the simulation of firm-specific trade disruption could consider resilience and vulnerability to trade disruption at the firm level and thus identify critical importer firms in supply chains. This is an interesting investigation because we find a large variation in the network topology among firms within the industry (Fig 3). However, the abovementioned topics are currently beyond the scope of this paper.

## Supporting information

S1 Appendix Supporting information. (PDF)
