# Supplementary material for: Disruption of international trade and its propagation through firm-level domestic supply chains: A case of Japan
Source: PLoS One. 2023 Nov 27;18(11):e0294574. doi: 10.1371/journal.pone.0294574 (PMC10681234; doi:10.1371/journal.pone.0294574)
Supplement: S1 Appendix — (PDF) [file pone.0294574.s001.pdf]

Disruption of International Trade and its Propagation through Firm-level Domestic Supply Chains : A Case of Japan

Supporting Information

Hiroyasu Inoue
Yasuyuki Todo

Materials

The Tokyo Shoko Research (TSR) data do not contain information on the sales of each firm to final consumers or the transaction volume of each supply chain link. We estimate the former by dividing the final consumption of each industrial sector, taken from the input–output (IO) table of Japan in 2015 [4], among all firms in the sector in proportion to their sales. In addition, we estimate the transaction volume of each link using the following algorithm. First, each supplier's total sales less sales to final consumers are tentatively divided among its clients in proportion to clients' sales. Second, the tentative volume of each supplier-client transaction is summed up within each pair of industries. Then, we adjust the interfirm transaction volume so that the sum of the estimated volume within any industry pair is equal to the actual volume taken from the IO table of Japan in 2015.

We combine the TSR data with trade information at the firm level taken from the Basic Survey of Japanese Business Structure and Activities (BSJ), using firm identification numbers for the BSJ that are also included in the TSR data. Because the number of firms in the BSJ data is substantially smaller than that in the TSR data, we have to ignore the imports and exports of small firms that are not included in the BSJ data. However, the total imports and exports of firms in the BSJ data are 47.7 and 83.0 trillion yen, respectively, whereas those of Japan in 2019 taken from the customs data are 78.6 and 76.9 trillion yen [3][1]. Therefore, we assume that the BSJ data cover most exports of Japanese firms. Most exporters may be included in the BSJ data because only productive and thus large firms can usually export [2, 1]. The total exports in the BSJ data exceed those in the customs data, possibly because the indirect exports of firms through traders are double counted as exports of both producers and traders in the BSJ data. In contrast, the imports in our data are undervalued because those of small and medium-sized enterprises (SMEs) are not included. In particular, imports from China in our data, totaling 8.55 trillion yen, are particularly undervalued compared with those in the BSJ data, totaling 18.5 trillion yen. The reason for this is possibly that numerous SMEs that import inputs rely on imports from China. In contrast, those imports from Asia except for China in our data are 13.5 trillion yen and relatively closer to those in the BSJ data, 18.9 trillion.

Results

Disruption of exports

Fig. 2 illustrates the simulation results for the disruption of exports to the world, while Fig. 3 shows the results by region. We find three notable differences between the effects of import and export disruption. First, the disruption of the imports of intermediate goods has a far greater impact on the total production of the economy than does disruption of the exports of final products. For example, reductions in imports

[1] We also use BSJ data for 2018 to check the overall trend in the data and the validity of the use of the data for 2019.

from the world and exports to the world by 80% for 2 months reduce value added production by 41 and 2.7 trillion yen, respectively (Fig. 2). Despite the larger effect of import disruption than of export disruption, the total annual imports in the data used in our simulation, 45 billion yen, are approximately half of the total annual exports, 81 billion yen. Moreover, the share of imports in the total value of intermediate goods, 2.8%, is substantially smaller than the share of exports in total production, 16.7%. Second, as the duration of export disruption is extended, the loss in value added production naturally increases. However, the ratio of the loss in value added to total value added production declines, although the loss ratio increases exponentially in the case of import disruption. For example, Fig. 2 indicates that when exports to the world are disrupted by 80% for four weeks, the total production of Japan declines by 3.7% during that period. When the duration becomes six weeks and two months, the rate of the loss in value added production declines to 3.3 and 3.2%, respectively. Finally, the loss ratio is closely proportional to the strength of disruption, i.e., the rate of reduction in exports. For example, when exports to the world are disrupted by 20, 40, 60, and 80% for two months, total production declines by 0.65, 1.3, 2.1, and 3.2%, respectively (inset of Fig. 2).

Differences between import and export disruption

The differences between import and export disruption are due to the following three reasons. First, the effect of import disruption, i.e., a reduction in the supply of inputs, can be partially absorbed by utilizing the inventory of disrupted inputs. Therefore, the effect of import disruption is initially quite small. In contrast, the effect of export disruption, i.e., a reduction in demand, cannot be absorbed but rather is aggravated by inventory usage. When exporting firms face the shrinkage of their exports, their demand for intermediate products also declines. Because exporters hold inventories of their intermediate goods, they use these inventories for decreased production and drastically reduce their number of purchases from their suppliers immediately after export disruption. As a result, the production of their suppliers declines substantially, leading to a large initial reduction in value added production relative to total production.

Second, although the initial effect of import disruption is alleviated by the use of input inventory, its effect is aggravated over time as inventory becomes exhausted. Moreover, the effect propagates downstream through supply chains because a decrease in the level of production of a firm results in a decline in the level of production of its clients. The propagation is gradual because of the input inventory held by clients but can be substantial after a certain amount of time. In contrast, how the effect of export disruption is influenced by inventory is the opposite. Once the excess inventories of intermediates are used shortly after export disruption, exporters purchase more intermediate goods from their suppliers, and thus, the rate of production loss declines in the long run compared to that immediately after disruption.

Finally, the effect of import disruption is “leveraged” or propagated to more firms as the shock proceeds downstream to clients through input shortages. However, there is no such leverage effect of export disruption because firms facing a reduction in demand directly or indirectly due to export disruption simply reduce their level of production by reducing demand. As a result, the rate of value added loss due to import disruption increases exponentially as the strength of disruption rises, while that due to export disruption is proportional to its strength.

We experiment with simultaneous import and export shocks (Fig. 4) to determine the outcome if the shocks occur at the same time. Although the total reductions are not simple summations of those obtained from import and export shocks independently, they are slightly smaller than the summations. As the effect of import disruption is far greater than that of export disruption, we focus on the former in the following analyses.

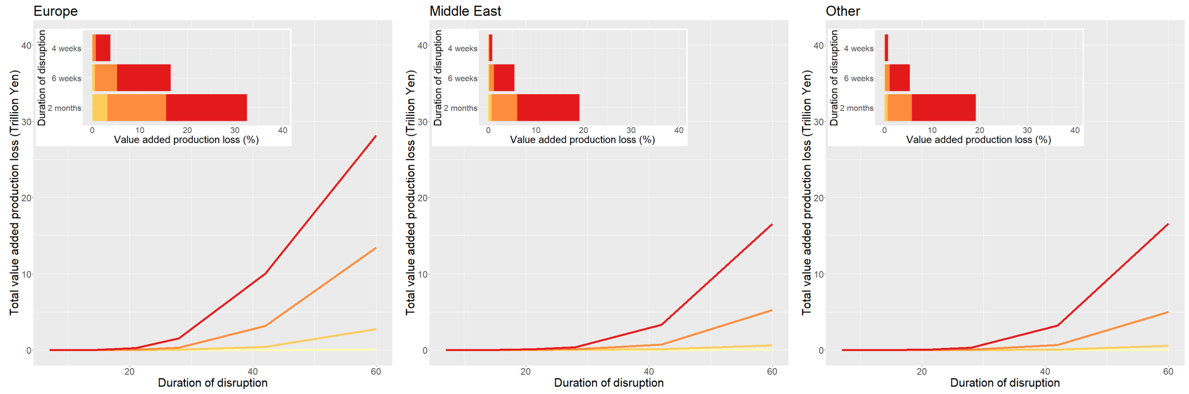

Figure 1: **Effect of the disruption of imports from various areas on domestic production.** The main panels in the figure show the total loss in value added production in Japan when imports from each region are disrupted to a particular extent (20-80%) for a particular duration (from 2 weeks to 2 months). The inset panels show the ratio of the loss in value added production to total production when imports from each region are disrupted at a particular strength for 4 and 6 weeks and 2 months. The right edge of each color of the bars indicates the loss rate at the corresponding strength (20, 40, 60, or 80%). These are supplements to Fig. 1 in the main text.

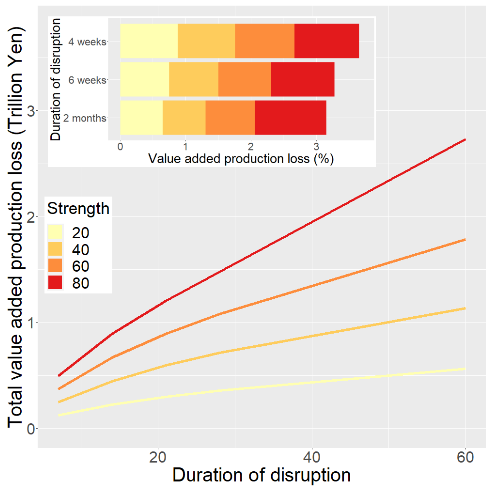

Figure 2: **Effect of the disruption of exports to the world on domestic production.** The main panels in the figure show the total loss of value added production in Japan when exports to the world are disrupted to a particular strength (20-80%) for a particular duration (from 2 weeks to 2 months). The inset panels show the ratio of the loss in value added production to total production when imports from and exports to the world are disrupted at particular strengths for 4 and 6 weeks and 2 months. The right edge of each color of the bars indicates the loss rate at the corresponding strength (20, 40, 60, or 80%).

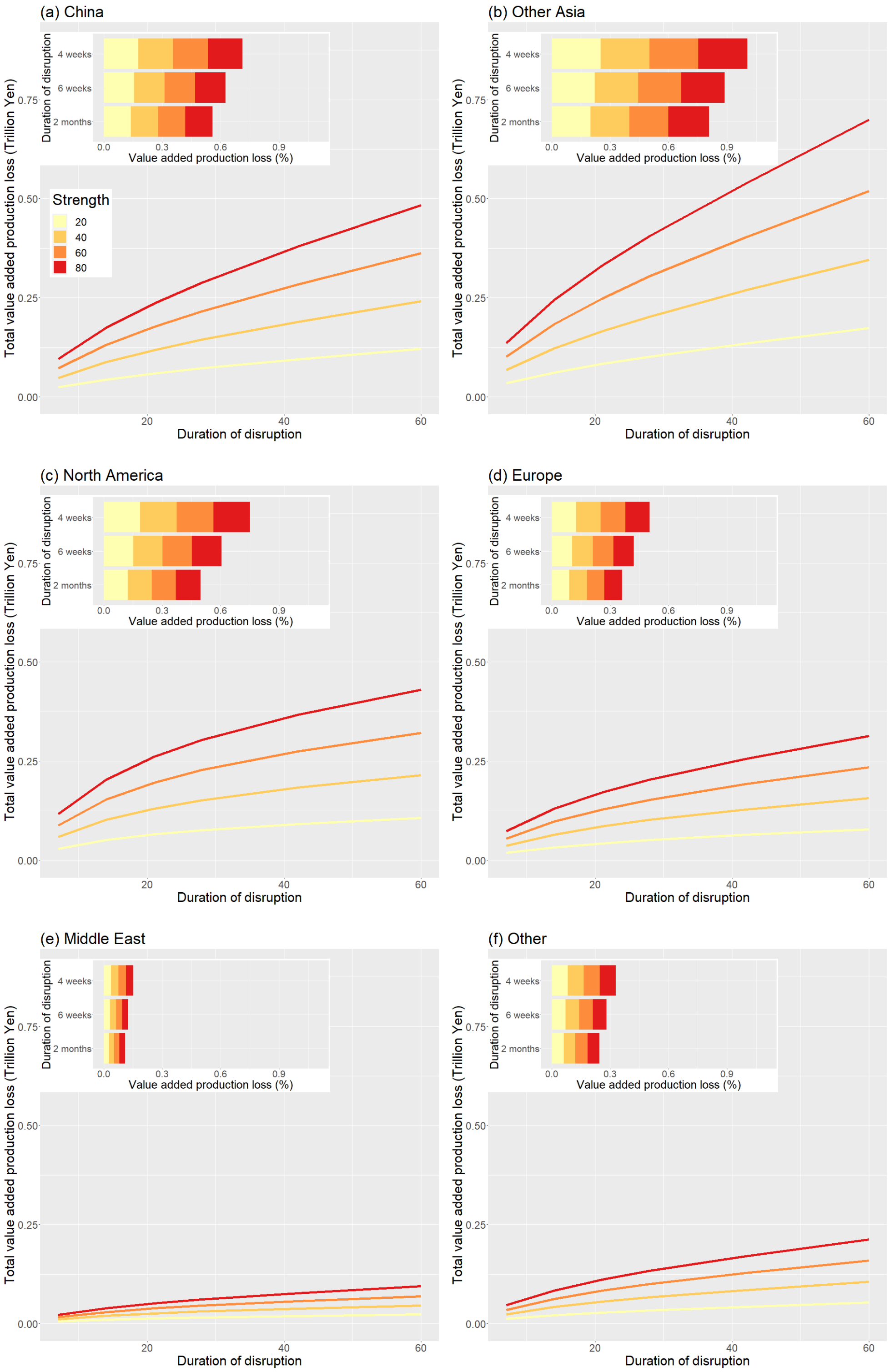

Figure 3: **Effect of the disruption of exports to various areas on domestic production.** The main panels in the figure show the total reductions in the level of production in Japan when exports to given areas are disrupted to a particular extent (20-80%) for a particular duration (from 2 weeks to 2 months). The inset panels show the ratio of the loss in value added production to total production when exports to each region are disrupted at a particular strength for 4 and 6 weeks and 2 months. The right edge of each color of the bars indicates the loss rate at the corresponding strength (20, 40, 60, or 80%). "Other Asia" represents Asian countries except for China.

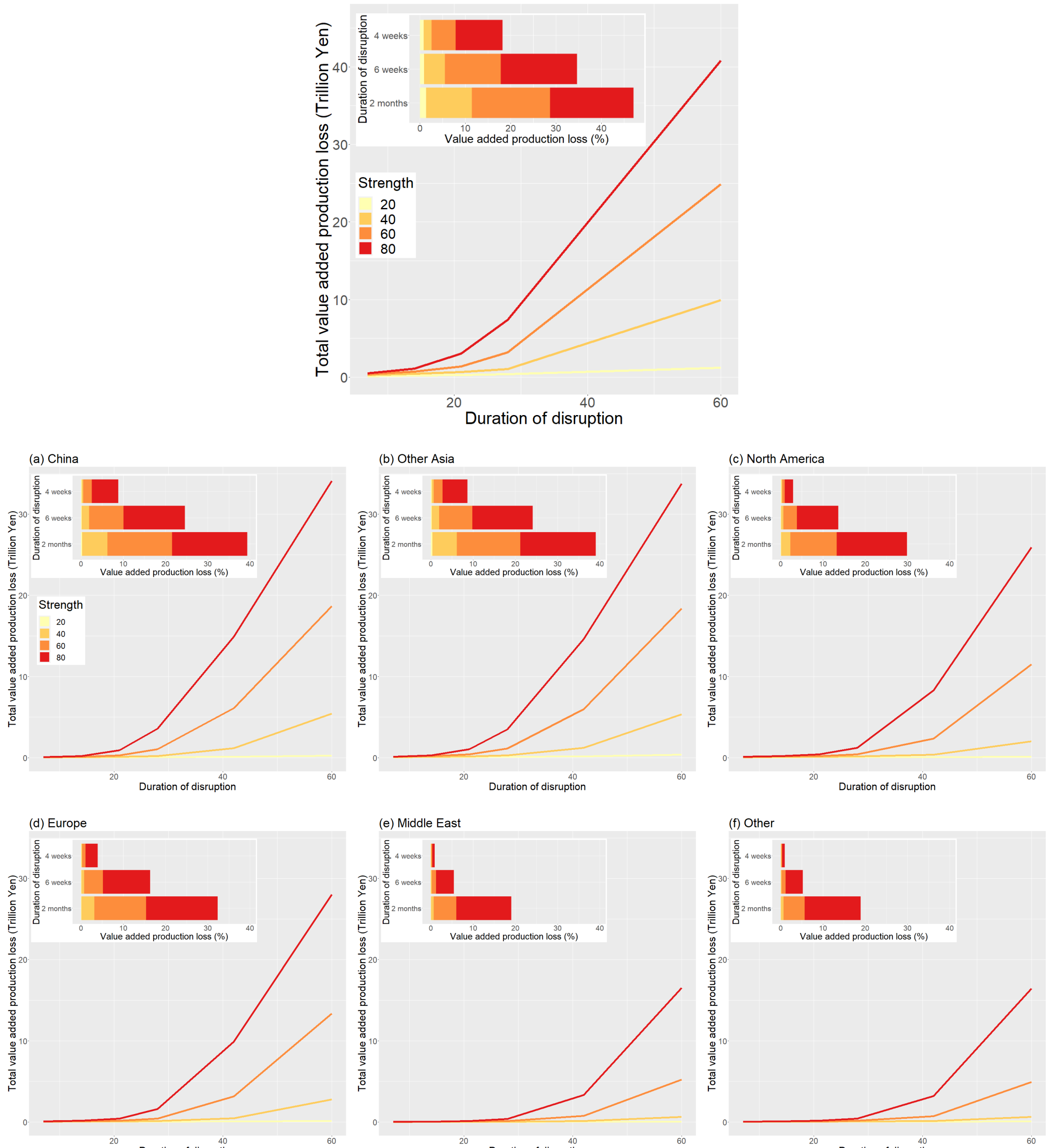

Figure 4: **Effect of the simultaneous disruption of imports and exports on domestic production.** The main panels in the figure show the total reductions in the level of production in Japan when the imports and exports of the world and specific areas are simultaneously disrupted to a particular extent (20-80%) for a particular duration (from 2 weeks to 2 months). The inset panel shows the ratios of the losses in value added production in Japan to total production when disruptions occur at a particular strength for a particular duration (4 and 6 weeks, and 2 months). Note that the bars are not cumulative, but the right edges of the bars indicate the rates of loss at the corresponding strengths. "Other Asia" represents Asian countries except for China.

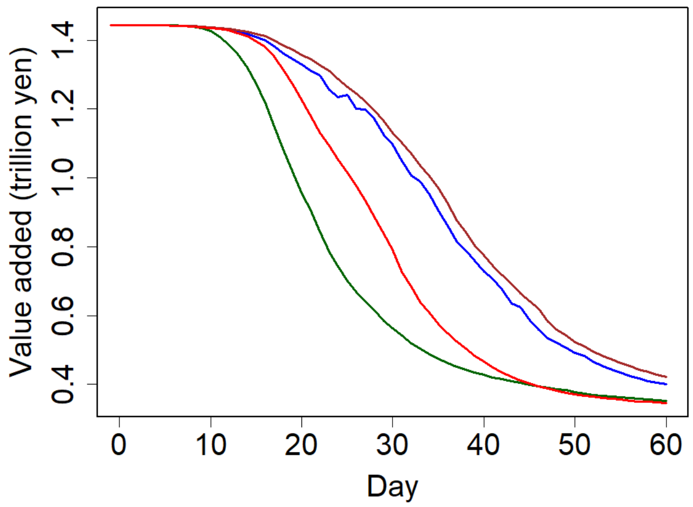

Figure 5: **Decrease in the level of production due to import disruption using different assumptions on the substitution of suppliers: The Chinese scenario.** This figure illustrates the total changes in daily value added after the disruption of imports from China by 80% for 60 days. Green, red, brown, and blue lines indicate changes assuming no supplier substitution, substitution between current suppliers in the same industry, perfect substitution with new suppliers, and substitution with new suppliers indirectly linked through supply chains, respectively.

Table 1: JSIC (Rev. 13, 2013) [5]: 1-digit level.

1-digit code	Industry
A	AGRICULTURE AND FORESTRY
B	FISHERIES
C	MINING AND QUARRYING OF STONE AND GRAVEL
D	CONSTRUCTION
E	MANUFACTURING
F	ELECTRICITY, GAS, HEAT SUPPLY AND WATER
G	INFORMATION AND COMMUNICATIONS
H	TRANSPORT AND POSTAL ACTIVITIES
I	WHOLESALE AND RETAIL TRADE
J	FINANCE AND INSURANCE
K	REAL ESTATE AND GOODS RENTAL AND LEASING
L	SCIENTIFIC RESEARCH, PROFESSIONAL AND TECHNICAL SERVICES
M	ACCOMMODATIONS, EATING AND DRINKING SERVICES
N	LIVING-RELATED AND PERSONAL SERVICES AND AMUSEMENT SERVICES
O	EDUCATION, LEARNING SUPPORT
P	MEDICAL, HEALTH CARE AND WELFARE
Q	COMPOUND SERVICES
R	SERVICES, N.E.C.
S	GOVERNMENT, EXCEPT ELSEWHERE CLASSIFIED
T	INDUSTRIES UNABLE TO CLASSIFY

Table 2: JSIC (Rev. 13, 2013) [5]: 2-digit level.

2-digit code for manufacturing industries	Industry
9	MANUFACTURE OF FOOD
10	MANUFACTURE OF BEVERAGES,TOBACCO AND FEED
11	MANUFACTURE OF TEXTILE PRODUCTS
12	MANUFACTURE OF LUMBER AND WOOD PRODUCTS, EXCEPT FURNITURE
13	MANUFACTURE OF FURNITURE AND FIXTURES
14	MANUFACTURE OF PULP, PAPER AND PAPER PRODUCTS
15	PRINTING AND ALLIED INDUSTRIES
16	MANUFACTURE OF CHEMICAL AND ALLIED PRODUCTS
17	MANUFACTURE OF PETROLEUM AND COAL PRODUCTS
18	MANUFACTURE OF PLASTIC PRODUCTS, EXCEPT OTHERWISE CLASSIFIED
19	MANUFACTURE OF RUBBER PRODUCTS
20	MANUFACTURE OF LEATHER TANNING, LEATHER PRODUCTS AND FUR SKINS
21	MANUFACTURE OF CERAMIC, STONE AND CLAY PRODUCTS
22	MANUFACTURE OF IRON AND STEEL
23	MANUFACTURE OF NON-FERROUS METALS AND PRODUCTS
24	MANUFACTURE OF FABRICATED METAL PRODUCTS
25	MANUFACTURE OF GENERAL-PURPOSE MACHINERY
26	MANUFACTURE OF PRODUCTION MACHINERY
27	MANUFACTURE OF BUSINESS ORIENTED MACHINERY
28	ELECTRONIC PARTS, DEVICES AND ELECTRONIC CIRCUITS
29	MANUFACTURE OF ELECTRICAL MACHINERY, EQUIPMENT AND SUPPLIES
30	MANUFACTURE OF INFORMATION AND COMMUNICATION ELECTRONICS EQUIPMENT
31	MANUFACTURE OF TRANSPORTATION EQUIPMENT
32	MISCELLANEOUS MANUFACTURING INDUSTRIES

Table 3: Overview of Japanese firms' imports to and exports from foreign areas.

Area	Import Volume (trillion yen)	# of links	Export Volume (trillion yen)	# of links
World	47.7	14,476	83.0	19,572
Asia	24.3	8,540	41.5	10,172
China	8.7	4,197	17.1	4,635
Other Asia	15.7	4,343	24.3	5,537
North America	4.6	2,257	19.0	3,339
Europe	5.2	2,575	12.4	2,951
Middle East	9.8	280	2.9	1,032
Other	3.8	824	7.2	2,078

Table 4: Regression results assuming import disruption for 28 days.

	(1)	(2)	(3)	(4)	(5)	(6)	(7)	(8)	(9)	(10)	(11)
ln_directshock	0.513***	0.962***	1.003***	1.002***	1.003***	1.029***	1.007***	0.666***	0.581***	0.618***	0.694***
	(0.109)	(0.105)	(0.119)	(0.120)	(0.120)	(0.117)	(0.119)	(0.119)	(0.107)	(0.109)	(0.138)
ln_importer	1.265***							0.818***			
	(0.228)							(0.282)			
ln_outdegreemean		1.435***						1.479**			
		(0.508)						(0.565)			
ln_indegreemean			0.932*					-0.487			
			(0.481)					(0.563)			
betweennessmean				4,998				11,757			
				(4,644)				(7,161)			
potentialmean					0.0809			1.877*			
					(1.360)			(1.028)			
ln_loopflowsummean						-1.400***		-0.869**			
						(0.320)		(0.405)			
clusteringmean							-4.253	-0.304			
							(7.443)	(6.316)			
ln_outdegreesum									1.004***		
									(0.212)		
ln_indegreesum										0.983***	
										(0.224)	
ln_betweennesssum											0.564***
											(0.176)
Constant	-10.65***	-9.704***	-9.157***	-8.147***	-8.144***	-1.681	-7.894***	-7.388**	-10.96***	-10.91***	-1.415
	(0.928)	(0.954)	(1.003)	(0.693)	(0.705)	(1.656)	(0.870)	(2.935)	(1.009)	(1.085)	(2.035)
Observations	110	110	110	110	110	110	110	110	110	110	110
R-squared	0.589	0.520	0.481	0.465	0.464	0.548	0.467	0.648	0.598	0.575	0.528

Robust standard errors are in parentheses.
*** p<0.01, ** p<0.05, and * p<0.1.

Table 5: Regression results assuming import disruption for 42 days. The results in Column (8) of this table are used in Fig. 10.

	(1)	(2)	(3)	(4)	(5)	(6)	(7)	(8)	(9)	(10)	(11)
ln_directshock	0.394***	0.865***	0.891***	0.901***	0.908***	0.938***	0.907***	0.569***	0.472***	0.455***	0.591***
	(0.124)	(0.126)	(0.127)	(0.136)	(0.135)	(0.133)	(0.135)	(0.137)	(0.123)	(0.115)	(0.147)
ln_importer	1.311***							0.856***			
	(0.241)							(0.306)			
ln_outdegreemean		1.339**						1.072			
		(0.542)						(0.711)			
ln_indegreemean			1.543**					0.534			
			(0.653)					(0.865)			
betweennessmean				3,850				11,582			
				(4,751)				(7,704)			
potentialmean					1.900			3.399***			
					(1.557)			(1.173)			
ln_loopflowsummean						-1.401***		-0.801*			
						(0.368)		(0.453)			
clusteringmean							-5.559	-0.590			
							(7.095)	(6.725)			
ln_outdegreesum									1.022***		
									(0.226)		
ln_indegreesum										1.117***	
										(0.253)	
ln_betweennesssum											0.566***
											(0.179)
Constant	-7.455***	-6.330***	-6.501***	-4.869***	-5.345***	1.554	-4.548***	-5.555*	-7.724***	-7.953***	1.882
	(1.041)	(1.085)	(1.238)	(0.794)	(0.848)	(1.871)	(0.964)	(3.342)	(1.142)	(1.237)	(2.061)
Observations	112	112	112	112	112	112	112	112	112	112	112
R-squared	0.485	0.405	0.405	0.360	0.374	0.441	0.363	0.568	0.490	0.498	0.420

Robust standard errors are in parentheses.
*** p<0.01, ** p<0.05, and * p<0.1.

Table 6: Regression results assuming import disruption for 60 days.

	(1)	(2)	(3)	(4)	(5)	(6)	(7)	(8)	(9)	(10)	(11)
ln_directshock	0.291**	0.656***	0.678***	0.687***	0.695***	0.712***	0.692***	0.391**	0.338***	0.311***	0.442***
	(0.125)	(0.133)	(0.132)	(0.141)	(0.139)	(0.141)	(0.140)	(0.158)	(0.125)	(0.114)	(0.156)
Number of importers (log)	1.026***							0.738**			
	(0.238)							(0.300)			
Average outdegree (log)		1.168**						0.894			
		(0.556)						(0.577)			
Average indegree (log)			1.552**					0.820			
			(0.704)					(0.611)			
Average betweenness				4,193				10,049			
				(5,092)				(11,787)			
Average potential					2.077			3.335***			
					(1.552)			(1.174)			
Average loop flows (log)						-0.920**		-0.371			
						(0.372)		(0.384)			
Average clustering coefficient							-3.794	1.465			
							(6.424)	(6.438)			
ln_outdegreesum									0.833***		
									(0.237)		
ln_indegreesum										0.944***	
										(0.266)	
ln_betweennesssum											0.449**
											(0.173)
Constant	-3.722***	-2.974**	-3.343**	-1.703**	-2.223**	2.521	-1.476	-4.317*	-4.025***	-4.305***	3.659*
	(1.096)	(1.175)	(1.344)	(0.833)	(0.894)	(1.933)	(0.965)	(2.381)	(1.242)	(1.335)	(2.045)
Observations	112	112	112	112	112	112	112	112	112	112	112
R-squared	0.348	0.296	0.311	0.255	0.275	0.297	0.257	0.436	0.360	0.375	0.301

Robust standard errors are in parentheses.
*** p<0.01, ** p<0.05, and * p<0.1.

References

[1] Andrew B. Bernard and J. Bradford Jensen. Why some firms export. *Review of Economics and Statistics*, 86(2):561–569, 2004.

[2] Marc J. Melitz. The impact of trade on intra-industry reallocations and aggregate industry productivity. *Econometrica*, 71(6):1695–1725, 2003.

[3] Japan Ministry of Finance. Trade statistics of Japan. `https://www.customs.go.jp/toukei/info/index_e.htm`, 2022. Accessed: 04.23.2022.

[4] Ministry of Internal Affairs and Communications, the Cabinet Office, the Financial Services Agency, the Ministry of Finance, the Ministry of Education, Culture, Sports, Science and Technology, the Ministry of Health, Labour and Welfare, the Ministry of Agriculture, Forestry and Fisheries, the Ministry of Economy, Trade and Industry, the Ministry of Land, Infrastructure, Transport and Tourism, and the Ministry of Environment, Japan. 2015 Input-Output tables for Japan. `https://www.soumu.go.jp/english/dgpp_ss/data/io/index.htm`, 2015.

[5] Ministry of International Affairs and Communications. *Japan Standard Industrial Classification (Revision 13)*, 2013. (accessed April 19, 2022).
